# Coachability: A Longitudinal Curriculum to Promote Medical Students’ Growth Mindset, Feedback Utilization, and Resilience

**DOI:** 10.15766/mep_2374-8265.11450

**Published:** 2024-10-11

**Authors:** Gabriella Tison-Brandon, Mary Dickerson, Laryn Sapetti, Raquel Johnson, Anna T. Cianciolo

**Affiliations:** 1 Second-Year Resident, Child Neurology, University of Louisville School of Medicine; 2 Second-Year Resident, Dermatology, Southern Illinois University School of Medicine; 3 Second-Year Resident, Family Medicine, Northwestern Medicine at Delnor Hospital; 4 Second-Year Resident, Physical Medicine and Rehabilitation, University of Missouri School of Medicine; 5 Professor, Medical Education, Southern Illinois University School of Medicine

**Keywords:** Coachability, Mindset, Resilience, Communication Skills, Feedback, Well-Being/Mental Health

## Abstract

**Introduction:**

The concept of medical student coachability, adapted from athletics and business management, offers a framework for characterizing students’ roles as clinical learners. We defined coachability as effectively seeking, receiving, and using feedback—even negative feedback—to change behavior and reach learning goals. To facilitate success in our clinical clerkships, we sought to empower preclerkship students’ capacity to be coached.

**Methods:**

Our curriculum comprised two large-group presentations and three small-group seminars totaling approximately 5 hours, distributed over 2 years: a year 1 orientation, a year 2 refresher, and a longitudinal year 2 seminar series. Medical students designed and first implemented the curriculum under faculty supervision in academic year (AY) 2015–2016 and have continuously managed and run it since. The AY 2022–2023 curriculum management team evaluated the curriculum cross-sectionally via student survey and focus groups.

**Results:**

Approximately 575 students have completed the curriculum since 2015. Immediately following curriculum delivery, AY 2022–2023 year 2 students (response rate: 70%-97%) rated it a valuable educational experience and described plans to implement the lessons learned in their clerkship. Focus group participants (eight clerkship students who participated in the coachability curriculum in AY 2021–2022) reported using coachability strategies to positive effect for their clinical learning and well-being.

**Discussion:**

Our curriculum's flexible, modular format facilitates adoption by others. Future development could expand coachability offerings across the continuum of medical school. However, the curriculum should remain led by students passionate about medical education and willing to try new things to continuously adapt content and instructional strategies.

## Educational Objectives

By the end of this curriculum, participants will be able to:

1. Articulate the value to coachability of a growth mindset, self-assess their own mindset, and articulate how to balance a performance mindset with a growth mindset to develop coachability.
2. Identify constructive feedback—including from patients—and establish techniques for eliciting and using constructive feedback.
3. Recognize burnout and its causes, including interpersonal conflict; self-assess their own burnout; and identify healthy strategies for coping with stress.

## Introduction

*Coachable* has been defined as “capable of being easily taught and trained to do something better.”^1^ In athletics, coachability is described as personal characteristics reflecting motivation, intensity of effort, openness to learning, respect for and trust in coaches and the coaching process, responsiveness to feedback, and ability to cope with criticism, setbacks, and change.^2,3^ Business management research supports this, as well as asserting the importance of goal striving and self-insight.^4–6^ We define coachability as effectively seeking, receiving, and using feedback—even negative feedback—to change behavior and reach learning goals.

Developing trainees’ coachability typically is seen as a task for the coach, rather than for the athlete or employee (though see Osman, Lane, and Goldsmith^6^). Training for clinical coaches emphasizes relational behaviors such as collaborative goal setting with learners, reciprocal feedback conversations, and joint strategizing for performance improvement,^7–10^ in line with the educational alliance^11^ concept. A relational coaching model is especially well suited to clinical instruction, where learning is best accomplished through participation in clinical work with guidance from more experienced members of the community of clinical practice.^12^ Though the educational alliance concept has been framed for teachers, it implies an important opportunity for learners to nurture effective learning relationships. Coachability offers a framework for characterizing how this role might function.

When our school's clerkship curriculum underwent reform to prioritize clinical immersion and socialization into medicine via coaching relationships with preceptors,^13,14^ curriculum designers—including medical students—identified student coachability as an important developmental target. To facilitate success in our clerkships, in which coaching relationships are short-term, we sought to empower students to develop their own capacity to be coached. As well as resources on faculty coaching, *MedEdPORTAL* has numerous publications that address individual aspects of medical student coachability, including receipt and use of feedback,^15,16^ managing interpersonal conflict,^17^ and resilience.^18^ Our curriculum offers an approach to developing these skills and attitudes holistically under the rubric of becoming coachable in the clinical setting. In addition, our curriculum was developed by students for students, enabling learners to take an active role in educational leadership and curriculum delivery. This publication presents our coachability curriculum as it exists today and has been recently evaluated cross-sectionally via student survey and focus groups.

## Methods

### Curriculum Overview

A group of medical students designed this curriculum by taking a literature- and stakeholder-informed approach to identify a set of coachability behaviors.^19^ First, the team documented the coachability behaviors our curriculum committees gave to clerkship coaches as performance targets: sets learning goals, asks questions, actively incorporates feedback, and adapts behavior to feedback. Next, the team examined the literature on constructs related to being coachable, such as self-regulated learning, feedback receptivity, and coping skills. Ultimately, the team adapted Butler and Winne's^20^ integrative analysis of feedback and self-regulated learning to create a learner-centered model of coachability. The coachability curriculum was first implemented as a series of three small-group seminars for year 2 students in academic year (AY) 2016–2017, the impact of which has been summarized elsewhere.^21^ This required curriculum (per our Educational Policy Committee) has remained student managed and run continuously since, and it has been adapted each year to meet students’ needs while staying consistent with our coachability model.

The current coachability curriculum, deliverable online or in-person, is ideally suited for medical schools with an active-learning culture and robust student engagement in medical education. In AY 2022–2023, the curriculum management team comprised four fourth-year students: one chair, who handled logistics, curriculum evaluation, and succession planning, and three content managers, who updated curriculum materials. Curriculum delivery was executed by the management team in collaboration with volunteer student presenters. Curriculum presenters were required to be part of our school's student-led medical education interest group, to have completed the coachability curriculum, and to be a third- or fourth-year in good standing. The management team coordinated with a medical education faculty supervisor via regular email updates, shared documents (Google Drive folder), and occasional brainstorming meetings.

AY 2022–2023 curriculum delivery consisted of two large-group PowerPoint presentations (one delivered during year 1 orientation, the other during year 2 orientation) and three small-group seminars delivered in concert with ongoing collaborative (problem-based) year 2 learning sessions. Large-group presentations took place in a lecture hall, using audiovisual equipment. The seminars took place in our problem-based learning tutor rooms, which included a table and seating for up to eight, a computer with a large-screen monitor, and a whiteboard. Prior to each seminar, the management team held a development session to alert student facilitators to any curriculum updates made since they had participated as learners and to orient them to their new role as near-peer instructors.

### Year 1: Foundations of Coachability

Delivered during the AY 2022–2023 year 1 orientation, this session consisted of a 60-minute interactive PowerPoint presentation, Foundations of Coachability (Appendix A). The presenter (a member of the coachability curriculum management team) introduced the concept of coachability by asking students about their experiences being coached before. She explained that the coachability curriculum was developed by medical students who realized that being coachable was a valuable skill across the continuum of medical education. Next, students filled out a locally adapted self-assessment of their mindset (growth vs. performance; Appendix B), and the presenter prompted them to reflect on their self-assessed mindset and when each mindset might be beneficial. Next, she introduced goal setting and how different types of goals reflected either a growth or a performance mindset. Students reviewed example goals and judged which mindset they reflected.

### Year 2: Exercising Coachability Skills

During the AY 2022–2023 year 2 orientation, a member of the management team delivered a 30-minute large-group PowerPoint presentation (Appendix C, Coachability Refresher). She reviewed the topics from year 1: coachability defined, balancing the growth and performance mindsets, and mindset-oriented goal setting. She also gave a brief overview of the upcoming seminars, encouraging students to keep an open mind and prepare themselves to participate. All seminars were conducted in AY 2022–2023, approximately 2 months after the refresher session and 2 months apart from each other. Third- or fourth-year medical students, including members of the curriculum management team, facilitated each seminar in 12 groups of five to seven students each.

Seminar 1: Feedback (Appendix D) focused on strategies to give and receive feedback. Facilitators asked students to participate in drawing activities, in which one participant served as the student and another as the coach, with each party receiving divergent, hidden instructions. Through this student–coach interaction, students discussed how differing expectations and miscommunication could produce ineffective feedback. Students tried to correct miscommunications and discussed how to handle feedback perceived as unhelpful or inaccurate.

Seminar 2: Conflict Resolution and Resiliency (Appendix E) focused on identifying signs of burnout, reflecting on interpersonal conflict as a contributor to burnout, and strategizing ways to resolve conflict as well as reduce burnout. Students participated in several activities, including completion of a modified version of the Maslach Burnout Inventory–Student Survey,^22^ reflection on an example conflict between a resident and a medical student, and self-identification of potential stressors and coping mechanisms.

Seminar 3: Patients as Coaches (Appendix F) focused on receiving, understanding, and reacting to challenging feedback from disgruntled patients in real time. Students participated in three different role-play scenarios that reflected difficult yet common situations medical students faced during clerkships. Two students volunteered to play either the patient or the student doctor in each scenario. Each scenario exercised different approaches to the same skills: responding to the patient in a calm manner while monitoring and maintaining the safety of the environment.

### Curriculum Evaluation

Beginning in AY 2018–2019, evaluation of large-group presentations comprised student opinions gathered via survey questions administered by year 1 and year 2 curriculum coordinators. These questions constituted a small part of the overall survey evaluation of the daylong year 1 and year 2 orientations. The orientation evaluation surveys were voluntary, did not collect identifiable information, and were not consistently administered every year. One close-ended, coachability-specific question asked students to rate the large-group presentation's value as a learning experience. One open-ended question asked students to provide general comments.

Survey evaluation of the AY 2022–2023 seminars (Appendix G) asked students to report (in open-ended format) the lessons taken away from each seminar, the planned application of those lessons, and any recommended seminar improvements. Students were also asked to rate the seminar content on four criteria (perceived importance, learning value, engagement, and whether or not they would recommend the session to others). The current curriculum management team developed the survey questions locally and administered the survey once following each seminar. The surveys did not gather personally identifiable information.

Also in AY 2022–2023, one curriculum manager conducted two in-person focus groups to assess the curriculum's utility to year 3 clerkships (Appendix H). Focus groups consisted of eight volunteer third-year students, recruited by email, who had completed the seminars previously (AY 2021–2022). Participants were unpaid; however, the curriculum's faculty supervisor—who also attended the focus groups—provided dinner. Of the eight focus group participants (four in each group), four identified as women, and one identified as underrepresented in medicine.

The facilitator began the focus groups by briefly revisiting the coachability curriculum, then asked the group members for their general impressions and observations on how the coachability curriculum had helped them in clerkship (Appendix H). The facilitator prompted students to provide specific examples of feedback difficulties, interpersonal conflict, or burnout experiences and what they had done to resolve these situations. We did not record the sessions, but the facilitator and the faculty supervisor took detailed notes, which they compiled and examined using collaborative interpretation.^23^

## Results

### Large-Group Presentation Survey Data

Tables 1 and 2 summarize student ratings of the year 1 and year 2 large-group presentations.

**Table 1.**
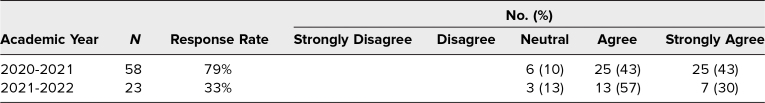
Student Ratings of the Year 1 Large-Group Presentation (Foundations of Coachability) as a Valuable Learning Experience

**Table 2.**
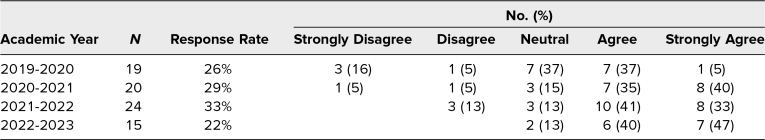
Student Ratings of the Year 2 Large-Group Presentation (Coachability Refresher) as a Valuable Learning Experience

Open-ended comments on the year 1 presentation (Foundations of Coachability) supported the ratings; students felt the presentation was educational and useful. One student stated, “It demonstrated that we aren't going to come in or leave perfect, but if our focus is on improvement… we will be more amenable to criticism and growth.”

Comments on the year 2 presentation (Coachability Refresher) suggested the content was appropriate and nicely recapped topics discussed the previous year. One student stated,

This session served as a good reminder of how to give and receive feedback. I appreciate how it emphasized how feedback is going to be a part of our journeys not only in medical school but later in practice as well.

Most critical comments noted that because this presentation was a recap, it did not include much new information.

### Seminar Survey Data

Table 3 summarizes the AY 2022–2023 seminar evaluation survey data. Sixty-nine students completed the curriculum, and the response rate ranged from 70% to 97%. Students generally agreed or strongly agreed that each session was important, valuable, engaging, and recommendable to others. However, seminar 2 received dissenting ratings from 2% to 8% of students.

**Table 3.**
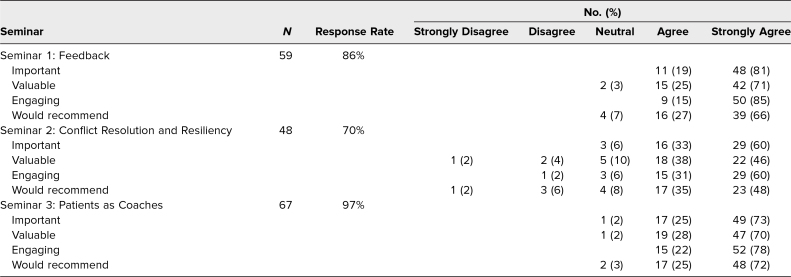
Student Ratings of the Year 2 Seminars

Consistent with the curriculum objectives for seminar 1 (Feedback), year 2 students reported finding value in clarifying expectations and setting goals (Table 4). Students also expressed increased confidence navigating feedback and voiced the value of seeking feedback specificity. Suggestions for improving the seminar included incorporating more diverse exercises (besides drawing), having slides to revisit later, and having specific examples of feedback to ask for in each clerkship.

**Table 4.**
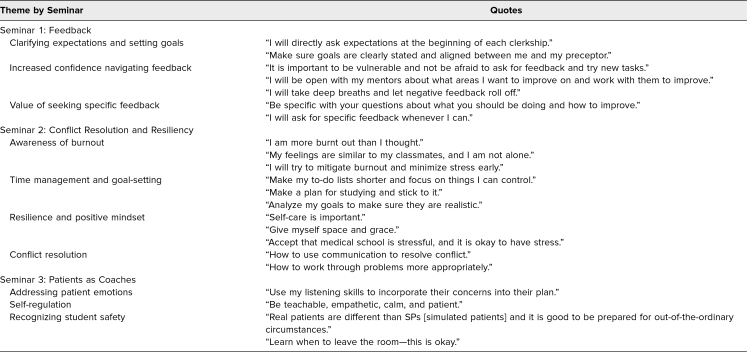
Thematic Contents of Open-ended Survey Comments on the Year 2 Seminars

Students’ comments regarding seminar 2 (Conflict Resolution and Resiliency) described improved awareness of burnout, reported plans to improve time management and goal setting, and identified the value of cultivating resilience and a growth mindset (Table 4). Suggestions for improving the session included allowing more time for discussion, adding more interactive questions and activities, adding a similar seminar to year 1, tailoring scenarios more to year 2, and shortening the seminar.

The student comments on seminar 3 (Patients as Coaches) emphasized acknowledging patient emotion while focusing on education and treatment (Table 4). Comments centered on maintaining one's own emotional composure while allowing oneself to be empathetic to patient concerns. Suggestions included adding more scenarios common for a medical student to face during third-year clerkships. Another suggestion recommended designing a seminar for de-escalation techniques and having more opportunities to discuss the scenarios throughout year 2.

### Focus Group Data: Seminars’ Impact on Clerkship

When focus group participants were asked to reflect on how the year 2 seminars helped them during clerkship, their report was consistent with students’ responses to the open-ended survey questions. Overall, students reported that after participating in the feedback seminar (seminar 1 in AY 2021–2022), they were more cognizant of the importance of constructive feedback and became vigilant in obtaining it. For example, they observed that this seminar prompted them to clarify expectations before a rotation's start to open the dialogue early. Students also reported learning to be mindful of when and where they asked a preceptor for feedback to improve the likelihood of having a full discussion with specific critiques. When one student received vague feedback (“good job”) on a hospital note they had written, they returned to the note later to infer from the preceptor's edits what improvements could have been made.

Although students did not as easily recall the conflict resolution seminar (seminar 2 in AY 2021–2022), they did reflect on ways it helped them in their clerkships. Students stated that they entered their clerkships understanding the importance of having someone available to debrief observed and experienced interpersonal conflicts. Students also commented that they most often encountered interpersonal conflict in the context of feedback. One student shared a situation where they received positive verbal feedback from a faculty member face-to-face, yet received negative written feedback. This student reported discussing this inconsistency with the faculty member and a supportive mentor.

Students reported learning many signs of burnout from the resiliency seminar (seminar 3 in AY 2021–2022). They cited several aspects of their clerkships that contributed to the burnout they were currently feeling and were able to list methods and resources they used to counter it. Students reported using several stress-reduction strategies discussed in the seminar, including conversations with supportive faculty, therapy, exercise, and perspective shifting.

## Discussion

Our coachability curriculum aimed to empower clerkship students with the capacity to cultivate effective coaching relationships. This capability is especially important in clerkships that use a relational coaching model yet feature short-term preceptor-student assignments. To design our curriculum, we developed a learner-centered model of coachability, and we have stayed true to this model while adapting the curriculum in response to changing student needs and curricular conditions. Our curriculum's theoretical grounding and flexible implementation bode well for wider adoption; the curriculum is well suited to facilitate learning in any clerkship that emphasizes coaching relationships, and its modular format allows responsiveness to evolving conditions. Our recent evaluation suggests not only that students found the curriculum valuable but also that they appeared to develop attitudes and behaviors reflecting coachability.

Our curriculum's flexibility also presents challenges; curriculum updates can require a year to pilot and a second year to make refinements. In AY 2022–2023, seminar 2 (Conflict Resolution and Resiliency) combined what had previously been two separate seminars to make space for a new addition (seminar 3: Patients as Coaches). Although our focus group students reported finding both the AY 2021–2022 resilience and conflict resolution seminars useful to clerkship, the combined session was least favored by AY 2022–2023 students. In addition, while most AY 2022–2023 students identified lessons learned about growth mindset, time management/goal setting, and burnout/resilience, few students mentioned learning lessons about conflict resolution. Positive perceptions of the new Patients as Coaches seminar reassure us that we should retain it in the future, but troubleshooting is needed to determine how best to balance these important topics without overloading students.

Survey questions on the year 1 and year 2 presentations had lower response rates and greater variation in ratings than the seminar surveys. However, these questions were incorporated into voluntary year 1 and year 2 orientation evaluation surveys collected by curriculum administrators up to 1 month after the presentations took place. By contrast, seminar survey data were collected by the coachability curriculum facilitators immediately following required coachability sessions. In addition, the large-group presentation questions constituted a very small subset of the orientation evaluation surveys, whereas the seminar surveys were focused solely on the coachability curriculum. These data-collection conditions make it challenging to infer meaning from variation in students’ ratings of the presentations, which could be due to a cohort effect or the impact of COVID restrictions on curriculum delivery. Even so, we have considered ways to improve the coachability refresher by introducing new topics that will become relevant later in the year, specifically incorporating the modified Maslach Burnout Inventory. Our curriculum evaluation would have been strengthened by engaging the coachability management team directly in evaluating the large-group presentations. In addition, recent developments in coachability assessment^24^ could be used to evaluate development of coachability behaviors directly.

An essential feature of our curriculum is student engagement. The curriculum can be managed entirely by students passionate about medical education with the supervision of just one faculty member. Importantly, designing, running, and managing this curriculum offer medical students a valuable opportunity to lead as curriculum cocreators^21^ and facilitate their junior peers’ clinical learning experiences. In our experience, students seem to appreciate support and encouragement from a peer who was recently in their place, which is consistent with the peer-assisted learning literature.^25^

Some potential limits on the transferability of our curriculum should be noted. Our medical school is small (class size = 72) and internationally recognized for educational excellence, including student engagement.^26^ It is unclear how well the seminars would work on a larger scale at institutions that do not support student engagement in education or in curricula that lack opportunities for small-group collaborative learning. Furthermore, we do not recommend that this curriculum be faculty run, given the value of peer-to-peer interactions to student buy-in, but attention must be paid to succession planning and student facilitator development. Most of this curriculum can be (and has been) delivered entirely online in the event of restrictions on in-person learning. However, the interactive seminars work best when conducted in person.

There are several ways this curriculum could be further modified and expanded. For example, our year 1 takes place on a campus 3 hours away from years 2–4, which prohibits feasible delivery of senior student-led seminars. At a medical school situated on a single campus, it would be possible to add seminars to begin developing coachability earlier than we do. Distributing the seminars across 2 years would also allow (re)separation of resilience and conflict resolution to give each topic the time it deserves. To respond to student coachability needs across the continuum of medical education, sessions could also be developed for senior students. Adaptation to student needs and curricular conditions has been central to the success of our curriculum, and we aim to help students feel more confident, learn better, and succeed in any environment.

## Appendices


Year 1 - Coachability.pptxYear 1 - Self-Assessment.docxYear 2 - Coachability.pptxSeminar 1 - Facilitator Guide.docxSeminar 2 - Facilitator Guide.docxSeminar 3 - Facilitator Guide.docxPostseminar Survey.docxFocus Group Protocol.docx
*All appendices are peer reviewed as integral parts of the Original Publication.*

## References

[1] Coachable. Britannica Dictionary. Accessed September 11, 2024. https://www.britannica.com/dictionary/coachable

[2] Fry MD, Hogue CM, Iwasaki S, Solomon GB. The relationship between the perceived motivational climate in elite collegiate sport and athlete psychological coping skills. J Clin Sport Psychol. 2021;15(4):334–350. 10.1123/jcsp.2020-0002

[3] Favor JK. The relationship between personality traits and coachability in NCAA divisions I and II female softball athletes. Int J Sports Sci Coach. 2011;6(2):301–314. 10.1260/1747-9541.6.2.301

[4] Weiss JA, Outland N, Plummer G, Zervos L, Carmichael-Tanaka N, Kang B. The stable individual differences driving employee coachability behaviours. Int J Evid Based Coach Mentor. 2023;21(2):102–117. 10.24384/d24j-fh23

[5] Somià T, Lechner C, Pittaway L. Assessment and development of coachability in entrepreneurship education. Int J Manag Educ. 2024;22(1):100921. 10.1016/j.ijme.2023.100921

[6] Osman S, Lane J, Goldsmith M. Becoming Coachable: Unleashing the Power of Executive Coaching to Transform Your Leadership and Life. 100 Coaches Publishing; 2023.

[7] Bannister SL, Wu TF, Keegan DA. The clinical COACH: how to enable your learners to own their learning. Pediatrics. 2018;142(5):e20182601. 10.1542/peds.2018-2601 30327376

[8] Sargeant J, Lockyer JM, Mann K, et al. The R2C2 model in residency education: how does it foster coaching and promote feedback use? Acad Med. 2018;93(7):1055–1063. 10.1097/ACM.0000000000002131 29342008

[9] Sargeant J, Armson H, Driessen E, et al. Evidence-informed facilitated feedback: the R2C2 feedback model. MedEdPORTAL. 2016;12:10387. 10.15766/mep_2374-8265.10387

[10] Watsjold B, Zhong D. Clinical Coaching Cards: a game of active learning theory and teaching techniques. MedEdPORTAL. 2020;16:11042. 10.15766/mep_2374-8265.11042 33274297 PMC7703484

[11] Telio S, Ajjawi R, Regehr G. The “educational alliance” as a framework for reconceptualizing feedback in medical education. Acad Med. 2015;90(5):609–614. 10.1097/ACM.0000000000000560 25406607

[12] Egan T, Jaye C. Communities of clinical practice: the social organization of clinical learning. *Health (London)*. 2009;13(1):107–125. 10.1177/1363459308097363 19103718

[13] Klamen DL. Getting real: embracing the conditions of the third-year clerkship and reimagining the curriculum to enable deliberate practice. Acad Med. 2015;90(10):1314–1317. 10.1097/ACM.0000000000000733 25901873

[14] Klamen DL, Williams R, Hingle S. Getting real: aligning the learning needs of clerkship students with the current clinical environment. Acad Med. 2019;94(1):53–58. 10.1097/ACM.0000000000002434 30157091

[15] Mariani A, Schumann SA, Fromme HB, et al. Asking for feedback: helping learners get the feedback they deserve. MedEdPORTAL. 2015;11:10228. 10.15766/mep_2374-8265.10228

[16] Fromme HB, Mariani AH, Zegarek MH, et al. Utilizing feedback: helping learners make sense of the feedback they get. MedEdPORTAL. 2015;11:10159. 10.15766/mep_2374-8265.10159

[17] Gunasingha RM, Knudsen N, Scialla T, Shepherd A, Clay A. Vital conversations: an interactive conflict resolution training session for fourth-year medical students. MedEdPORTAL. 2021;17:11074. 10.15766/mep_2374-8265.11074 33511271 PMC7830754

[18] Bird A, Tomescu O, Oyola S, Houpy J, Anderson I, Pincavage A. A curriculum to teach resilience skills to medical students during clinical training. MedEdPORTAL. 2020;16:10975. 10.15766/mep_2374-8265.10975 33015355 PMC7526502

[19] Murray E, Fetter M, Ghareeb A, Cianciolo AT. Coachability: a student-led curriculum to promote clinical learning. Med Educ. 2018;52(11):1183–1185. 10.1111/medu.13712 30264409

[20] Butler DL, Winne PH. Feedback and self-regulated learning: a theoretical synthesis. Rev Educ Res. 1995;65(3):245–281 10.3102/00346543065003245

[21] Stoddard HA, Lee AC, Gooding HC. Empowerment of learners through curriculum co-creation: practical implications of a radical educational theory. Teach Learn Med. Published online February 8, 2024. 10.1080/10401334.2024.2313212 38332636

[22] Shi Y, Gugiu PC, Crowe RP, Way DP. A Rasch analysis validation of the Maslach Burnout Inventory–Student Survey with preclinical medical students. Teach Learn Med. 2019;31(2):154–169. 10.1080/10401334.2018.1523010 30577705

[23] Wassler JD, Bresler L. Working in the interpretive zone: conceptualizing collaboration in qualitative research teams. Educ Res. 1996;25(5):5–15. 10.3102/0013189x025005005

[24] Ober TM, Williams KM, Kell HJ, Holtzman S. Measuring coachability by situational judgment task: development and initial validation. Pers Individ Dif. 2024;219:112503. 10.1016/j.paid.2023.112503

[25] Loda T, Erschens R, Nikendei C, Zipfel S, Herrmann-Werner A. Qualitative analysis of cognitive and social congruence in peer-assisted learning—the perspectives of medical students, student tutors and lecturers. Med Educ Online. 2020;25(1):1801306. 10.1080/10872981.2020.1801306 32744892 PMC7482745

[26] Cianciolo AT, Klamen DL, Beason AM, Neumeister EL. ASPIRE-ing to excellence at SIUSOM [version 1]. MedEdPublish. 2017;6:82. 10.15694/mep.2017.000082 38406429 PMC10885253

