# Supplementary material for: Coachability: A Longitudinal Curriculum to Promote Medical Students’ Growth Mindset, Feedback Utilization, and Resilience
Source: MedEdPORTAL. 2024 Oct 11;20:11450. doi: 10.15766/mep_2374-8265.11450 (PMC11467082; doi:10.15766/mep_2374-8265.11450)
Supplement: Supplementary file 1 — Year 1 - Coachability.pptxYear 1 - Self-Assessment.docxYear 2 - Coachability.pptxSeminar 1 - Facilitator Guide.docxSeminar 2 - Facilitator Guide.docxSeminar 3 - Facilitator Guide.docxPostseminar Survey.docxFocus Group Protocol.docx [file mep_2374-8265.11450-s001.zip › F. Seminar 3 - Facilitator Guide.docx]

**Seminar 3 – Patients as Coaches – Facilitator Guide**

Seminar Objectives:

1. Articulate what coachability means and why it is important to be responsive to patient feedback
2. Describe how patient coachability differs from resident/attending/peer coaching
3. Identify ways to practice shared decision making, even if you do not agree with the patient’s perspective
4. Articulate how to make sure the patient feels heard despite the inevitable time constraints in medicine
5. Identify general strategies and exit plans for emotionally charged patient encounters

Seminar Preparation: This seminar comprises three semi-scripted role-play scenarios to help pre-clinical students prepare for learning from patient feedback during their clerkship rotations. This seminar is more complex than the previous two seminars, and we recommend reading this entire facilitator guide as the first preparatory step. After reviewing the guide, print enough scripts for each scenario (appended to the end of this guide) so that role-players do not share scripts.

Seminar Conduct: The seminar is ideally conducted in small groups of six students and is amenable to either in-person format (preferred) or virtual format. Including introductory and closing discussion, the seminar should last 50-60 minutes. Each scenario is supposed to last 10 minutes. In each scenario, one student will role-play a Patient/Parent and another student will role-play a Student, while the other members of the small group observe and provide input when asked. We recommend assigning different students to each role-play scenario so that everyone has a chance to participate actively. The general conduct of each role-play scenario should occur as follows:

1. Read the scenario instructions to the students.
2. Distribute the appropriate script to each of the two assigned role-players. (The Patient/Parent role-player receives only the Patient/Parent script. The Student role-player receives only the Student script. Students do not receive scripts for scenarios they are observing instead of role-playing.)
3. Give the role-players a chance to review their scripts and signal you when they are ready to begin.
4. As the role-play proceeds, listen for “cue phrases” from the Student role-player (provided for each scenario in the guidance below). These cue phrases signal that the Student role-player has addressed the practice points for the scenario. *Note, these cue phrases are not part of the Student role-player's script. They are to be listened for as the Student role-player reaches the end of the script and must improvise to complete the role-play interaction.* When you hear one of these cue phrases, end the role-play.
5. Note that there is also a cue phrase for the Patient/Parent role-player. This cue phrase is provided at the end of the Patient/Parent role-player's script, and it signals failure of the Student role-player to address the practice points for the scenario. The Patient/Parent role-player's script prompts the role-player to say this cue phrase if they have read through their entire script.
6. Student role-players may say “Time Out” to pause the interaction and ask their groupmates for help. Neither the facilitator nor the Patient/Parent role-player may assist during a Time Out, however the rest of the group should be encouraged to brainstorm ideas with the Student role-player. The Student role-player may say “Time In” when they are ready to proceed with the scenario.
7. There are three prompts to end each scenario:
   1. If a cue phrase is spoken by the Student or Patient/Parent role-player
   2. If you feel the scenario practice points (provided in the guidance below) have been addressed
   3. If the scenario has come to a standstill where the Student role-player does not know what to say, even with help from groupmates
   4. If the scenario has lasted past the allotted time
8. After the scenario, the Facilitator will guide group reflection and discuss the practice points for this encounter and the approaches role-players used.

Facilitator Instructions and Guidance:

**Seminar Part 1: The Meaning of Coachability and Coachability from a Patient Encounter Perspective** (10 min)

The first question to ask students is “What does Coachability mean to you? It is a term used superficially before and during clerkships but is rarely defined.” Listen to the students' answers and confirm the definition to be “The ability to be easily taught and trained to do something better.”

Ask the students for examples of how to be coachable during their clerkships. Listen to their answers and confirm several means of coachability, for example:

- Asking for feedback from residents and attendings
- Taking advice when it is offered
- Reviewing learned material that night and returning the next day with questions to further develop growth within the topic
- Asking questions and conveying interest during the rotation

Ask the students what it means to be a coach and who their coaches are as medical students. Follow by asking how to be coachable from patients as well as from other faculty and staff members. Listen to their answers and emphasize that this is different from receiving feedback from a preceptor. Specifically:

- Most coachability is guided by preceptors outside of patient encounters, but to be coachable by a patient requires awareness during the patient encounter to realize when to change your language, attitude, or approach. This is very difficult to do, but that is the purpose of this session.
- Because patient feedback must be analyzed after the patient interaction, a medical student being coachable from the patient usually means that the changes to the student's behavior cannot be implemented until after the interaction. They cannot typically be done in the moment.

**Seminar Part 2: The 3 Scenarios** (35 min)

Begin by reading the Part 2 Instructions below (5 min):

“In this part of the seminar, there are three scenarios that each require two role-players: one Student role-player and one Patient or Parent role-player. These scenarios are partially scripted, and at some point in each scenario the Student role-player will need to improvise.”

“During the scenario, Student role-players may say the phrase “Time Out” to pause the interaction and ask their groupmates for help navigating the situation. Neither I nor the Patient/Parent actor may assist during a Time Out, however the rest of the group is encouraged to brainstorm ideas with the Student role-player. I expect everyone to participate in this brainstorming process. The Student role-player may say “Time In” when they are ready to proceed with the scenario.”

"I will signal the end of each scenario and then there will be a group discussion about the practice points contained in the scenario and the approaches that were taken.”

*Scenario 1: HPV Vaccine* (10 min)

Request volunteers to serve as role-players in this scenario. Pass out the scripts to assigned role-player.

- The Parent role-player receives only the Parent script. The Student role-player receives only the Student script. Make sure the role-players keep their scripts to themselves and do not share them with each other or their groupmates.
- Give the role-players a chance to read their script and signal you when they are ready to begin.
- Allow the role-play to proceed, listening for cue phrases.

Student role-player cue phrases (NOTE - similar sounding phrases count as cue phrases)

- “This vaccine is the only vaccine against cancer that we have, and it would protect any kid who gets it.”
- “We are not supporting sex between minors, but creating a safety net just in case he is exposed at some point in his life.”
- “I must offer/mention this vaccine in every school physical for children his age. It is a part of my job to make sure you have all the information you need to make an informed decision for him.”

Parent role-player cue phrase (NOTE - similar sounding phrases count as cue phrases)

- “Is this vaccine mandatory for him to start school? Then I don’t want it!”

You may end the scenario if (1) you hear one of the above cue phrases; (2) you feel the interaction has addressed the scenario’s practice points below; (3) the Student role-player becomes stuck even with help from groupmates; or (4) the interaction has gone past the allotted time.

Next, foster group discussion on the scenario, asking students what they believe the practice points in this scenario were and what they took away from participating in it. Listen to their responses, then read the practice points.

The Scenario 1 practice points are:

- It is important to know how to field parental upset during a typical discussion about primary prevention.
- Not every patient or parent will be nice or calm. Keep your cool and attempt to recognize their point of view. Get your point across without offending or cutting ties with the parent.
- Trust between patient and provider is built over time with consistency and transparency. This includes telling them what you can offer and providing up to date information on the subject matter. It is important to explain this to the parent or patient (You may tell them the student actor cue phrases to supplement this discussion)
- Transparency and consistency are greatly appreciated by patients. If they do not like what you are saying, they can always refuse. You can still inform them if they refuse.

Next, review the cue phrases for this scenario, as listed above.

Finally, review the Big Takeaway at the end of the discussion to reiterate the point of this scenario. Please read the Big Takeaway out loud, and then answer the questions that arise at this point.

Big Takeaway

“These interactions with patients will come up over the course of your clerkship and throughout your career. The goal is to not chase the patient or parent away by arguing them down while also not being intimidated if their demeanor is less than ideal. Our job is to educate patients so that they can make an informed decision about their own healthcare (not to argue, judge, or force their hands). This is shared decision making and is a practice that will be used frequently. What questions do you have for me?”

*Scenario 2: Patient feels left out of their own care/unheard* (10 min)

Request volunteers to serve as role-players in this scenario. Pass out the scripts to assigned role-player.

- The Patient role-player receives only the Patient script. The Student role-player receives only the Student script. Make sure the role-players keep their scripts to themselves and do not share them with each other or their groupmates.
- Give the role-players a chance to read their script and signal you when they are ready to begin.
- Allow the role-play to proceed, listening for cue phrases.

Student role-player cue phrase (NOTE - similar sounding phrases count as cue phrases)

- “I hear your concerns, but I do have to move on to see other patients this morning. I will take your concerns to my attending, and we will discuss this later when we come back to your room later today.”

Patient role-player cue phrase (NOTE - similar sounding phrases count as cue phrases)

- “So, when am I going to get out of here?”

You may end the scenario if (1) you hear one of the above cue phrases; (2) you feel the interaction has addressed the scenario’s practice points below; (3) the Student role-player becomes stuck even with help from groupmates; or (4) the interaction has gone past the allotted time.

Next, foster group discussion on the scenario, asking students what they believe the practice points in this scenario were and what they took away from participating in it. Listen to their responses, then read the practice points.

The Scenario 2 practice points are:

- Listen to the story and hear the whole thing to grasp why the patient is so anxious. Your time constraints, which, as a student, are much more lax than that of residents. You have time to listen!
- This builds trust with patients as well as teaches you more about them beyond their diagnosis.
- Time to listen is not unlimited, so a polite, yet firm line reiterating that you have heard your patient, and you will be taking the information back to your superiors should be used.
- Try to make this reiteration as personal as you can. Include aspects of their history in it to let them know you were actually listening.

Next, review the cue phrases for this scenario, as listed above.

Finally, review the Big Takeaway at the end of the discussion to reiterate the point of this scenario. Please read the Big Takeaway out loud, and then answer the questions that arise at this point.

Big Takeaway

“These interactions with patients will come up over the course of your third year and throughout your career. The goal is not to hurry out of the room abruptly, leading to more patient anxiety, or to get caught in a patient ramble for longer than reasonable. We want to understand the patient as a person and how their illness is affecting them beyond the medical realm. We want to help people, not just their ailments. What questions do you have for me?”

*Scenario 3: Patient is angry at staff due to miscommunication* (10 min)

Request volunteers to serve as role-players in this scenario. Pass out the scripts to assigned role-player.

- The Patient role-player receives only the Patient script. The Student role-player receives only the Student script. Make sure the role-players keep their scripts to themselves and do not share them with each other or their groupmates.
- Give the role-players a chance to read their script and signal you when they are ready to begin.
- Allow the role-play to proceed, listening for cue phrases.

Student role-player cue phrase (please be aware that similar sounding phrases count as cue phrases)

- “I’m sorry, it sounds like we have altered your schedule. Let me grab my supervisor so we can get you out of here.”
- NOTE - Both the apology and excusing themselves from the room is necessary for this to end the scene.

Patient role-player cue phrase (please be aware that similar sounding phrases count as cue phrases)

- “I’m sorry, WHO are you?? A STUDENT? Get me a real doctor in here!!”

You may end the scenario if (1) you hear one of the above cue phrases; (2) you feel the interaction has addressed the scenario’s practice points below; (3) the Student role-player becomes stuck even with help from groupmates; or (4) the interaction has gone past the allotted time.

Next, foster group discussion on the scenario, asking students what they believe the practice points in this scenario were and what they took away from participating in it. Listen to their responses, then read the practice points.

The Scenario 3 practice points are:

- This patient’s feedback comes from both verbal language and body language. Reading a patient’s demeanor and body language while in the moment is a difficult task that can be built with more practice.
- Know the details of the patient’s situation (in this case, waiting longer than expected) and realize where their emotions are originating from.
- Listening to patient concerns/let them talk to improve patient compliance and decrease confusion. You should be able to recognize that this scenario is different from Scenario 2 due to the patient’s hostile nature.
- Do not bait or insult the patient. You may acknowledge their anger, i.e. “*I can see you’re upset, and I am very sorry this has taken so long.*”
- Lastly, an apology can go a long way. It may not be your fault, but the clinical system has failed this patient today and it is ok to apologize for that.
- Recognize a hostile environment and know when and how to excuse yourself appropriately. This is appropriate even if you have been sent in to complete a task like a medicine reconciliation. If you do not feel safe, you have the right to leave. When you recognize hostility, you should say as little as possible before excusing yourself with “*I am going to get my supervisor for you.*”

Next, review the cue phrases for this scenario, as listed above.

Finally, review the Big Takeaway at the end of the discussion to reiterate the point of this scenario. Please read the Big Takeaway out loud, and then answer the questions that arise at this point.

Big Takeaway

“Although the other two scenarios focused on listening to the patient and trying to decipher their messages, this scenario focused on completing a very specific task that did not align with the patient’s goals. This resulted in hostility from the patient toward the student. As a student, you are not responsible for such patients and assistance from your superiors is necessary. The big takeaway here is to recognize when you are in a hostile situation as opposed to a situation of misunderstanding and how to remove yourself from this situation. An apology can be powerful here. It may both establish a connection between you and the patient and offer you a moment to exit the situation. These are difficult skills and will come with more practice. What questions do you have for me?”

**Part 3: Closing and Questions** (10 min)

Facilitate group discussion on the closing questions below. Choose who answers these questions. Pick a different person to ask for every question. Allow others to share their opinions after the initially chosen person offers an answer.

1. What is coachability?
   1. “The ability to be easily taught and trained to do something better.”
2. Why do we want to be coachable from a patient perspective?
   1. Helping the patient is our purpose in medicine. While we go to school for many years to learn what it takes to do this down to a molecular level, patient health and satisfaction is our ultimate goal. Learning how to bridge the gap between our vast medical knowledge and the patient’s goals for their own care is essential to achieving these goals.
3. How can we be coachable from a patient perspective?
   1. Listening to patient concerns and trying to sympathize with them from
      their perspective.
   2. Reading patient body language to gauge emotions.
   3. Asking patients what their general knowledge on the subject is and what
      they want to know.
   4. Keeping a cool head if a patient’s emotions flare in order to
      provide them with the best care we can offer.
4. How should we respond to patients who are angry or frustrated?
   1. Try to listen and discern the problem. Be aware of their attitude and level of hostility because you may need to excuse yourself to go get back up.
5. How should we respond when the patient’s problems go beyond our scope as students?
   1. Try to listen and discern the problem, but do not be afraid to tell them that you do not know the answer while offering to grab your superior for more assistance.

Reiterate these points:

- *Being coachable from the perspective of patients is a skill developed over*
  *time. The more patients you see, the more practice you will get.*
- *Try to remain calm when seeing patients. You are the one with the knowledge*
  *who is here to help them with their care.*
- *Real people are not the same as simulated patients! Listening to and talking to them like*
  *real people along with providing information they need to make an informed*
  *decision builds trust right away.*
- *You have the support of your residents and preceptors, and you may consult them if*
  *you ever feel overwhelmed by a topic or patient.*

**Scenario Role-Play Scripts**

**Scenario 1**

**Role = Student Role-player**

Scene = Parent of an 11-year-old boy is here for Tdap and flu vaccines before school and the med student is told by their attending to bring up HPV.

Script

**Student Role-player (SR)** - “*Alright Mrs./Mr. Jones, I also see that Kevin has not had an HPV vaccine. Is that something you would be interested in today?*”

**Parent Role-player** - “*HPV? What’s that?*”

**SR** - “*Human papillomavirus (HPV) is a sexually transmitted infection that can lead to cervical cancer as well as genital warts and cancers of the mouth/throat, penis and anus.*”

At this point, please respond to the parent with the best of your knowledge.

**Scenario 1**

**Role = Parent Role-player**

Scene - Parent of an 11-year-old boy is here for Tdap and flu vaccines before school and the med student is told by their attending to bring up HPV.

Script

**Student role-player** - “*Alright Mrs./Mr. Jones, I also see that Kevin has not had an HPV vaccine. Is that something you would be interested in today?*”

**Parent role-player** - “*HPV? What’s that?*”

At this point, the student role-player will attempt to explain the vaccine. **You are to argue and cut off SA from here on out with at least 5 different argumentative phrases**. You may make up your own phrases or use the phrases provided below. Your arguments do not have to be tied together or even make sense. The goal here is to surprise the student doctor with interruptions and aggressive statements.

Argumentative lines used to cut off Student role-player:

- “*My son doesn't even have a cervix, and even if he did, why would I want to encourage him to have unsafe sex?”*
- “*My cousin’s brother’s neighbor’s friend who moved to Nevada said that this shot increased his daughter’s sex drive!*”
- “*We are very religious and don't believe in sex before marriage, so why should I care about HPV? My son will never be involved in such filth!*”
- “*Don’t try to push your agenda onto my son! Why would he get more shots than he needs? If it's such a good idea, why have I never heard of it?*”
- “*I have never had this so-called cancer shot and I’m doing just fine!! Why would I subject him to this??*

To signal the end of the scenario, you may end with “***Is this vaccine mandatory for him to start school? Then I don’t want it!***”

**Scenario 2**

**Role = Student Role-player**

Scene - Inpatient setting, 85-year-old patient has been getting poked and prodded since getting moved up from the ED after sitting there all night. Patient has not slept well due to their circumstances. Diagnosis is non-COVID pneumonia. It is now 7:30 am and a whole new team is arriving. The patient list is capped so the resident has to move quickly and only does a brief physical exam on the patient, leaving the medical student in the room to gather a more thorough history.

Script

**Student Role-player** - **“***Good morning! Sorry to wake you, I am the third-year medical student assisting with your care***”**

**Patient Role-player** - “Why hello, I have some questions for you if you don’t mind me asking.”

At this point, please respond to the patient with the best of your knowledge. You may use what has been learned in the previous scenario here.

**Scenario 2**

**Role = Patient Role-player**

Scene - Inpatient setting, the 85-year-old patient has been getting poked and prodded since getting moved up from the ED after sitting there all night. The patient has not slept well due to their circumstances. Diagnosis is COVID pneumonia. It is now 7:30 am and a whole new team is arriving. The patient list is capped so the resident has to move quickly and only does a brief physical exam on the patient, leaving the medical student in the room to gather a more thorough history.

Note - Be obviously anxious and constantly interrupt the Student role-player (SR) during their typical introduction with a seemingly unrelated line of questioning. Try to cut them off mid-sentence with another question. More interruptions will come the more SR tries to answer. You have a spouse to take care of waiting for you at home and don’t have time for doctor foolishness. You may deviate from the written lines but **be sure to constantly ask when you can leave the hospital.**

Script

**Student Role-player (SR)** - **“***Good morning! Sorry to wake you, I am the third-year medical student assisting with your care***”**

Argumentative lines you can use to cut off the SR:

- *“Who are you? What do you want?* ***When can I leave?”***
- *“Is breakfast here? What are they selling? Chocolates?* ***When can I go home?”***
- *“Why do I have an alarm on my bed? Where is my old nurse?* ***Is it time to go now?”***
- *“Why is my arm hurting? Does this bed get any more comfortable?”*
- *“Was it the chicken or the egg that came first?* ***Can I call my son to come get me?*”**

If the SR asks why you would like to go home, you may use some of the below lines to tell your character’s story. This portion is to be a sort of “word vomit” where you may read your lines without interruption. You may end at any time with the final line at the bottom of your script.

- “*Couldn't breathe last night and I didn’t even want to come here but my son made me even though I can't afford it since I have to stay home and take care of my Marty/Martha; that's my husband/wife you see?*”
- “*Marty/Martha has Parkinson’s and I take care of ‘em because that’s what you do when you love somebody. Our son has moved home to look after Marty/Martha, but cannot stay long; he has to get back to work and pay his own bills.*”
- “*Marty/Martha has real troubles getting to bathroom in the middle of the night. Seeing in the dark and carrying -em takes so much out of me*”
- “*Marty/Martha never the outdoorsy type. I can’t get -em to exercise for the life of me, only wants to sit in front of the tv and watch MASH or Wheel of Fortune or that doggone Steve Harvey on Family Feud.*
- “*Marty/Martha has had Parkinson’s for 15 years, I used to be able to lift them but now it is a little harder. But you gotta do what you gotta do*”

To signal the end of the scenario, you may end with ***the final line*** *-* ***“So when am I gonna get out of here?”***

**Scenario 3**

**Role - Student Role-player**

Scene - A long day in the clinic has gone longer than expected and a patient has been left waiting in a clinic room for over an hour. Due to the overload, the student has been sent in to see this patient on their own while their preceptor is handling a more complicated patient. Patient is here for a medication reconciliation and refill for her SLE which has flared up. They are now late for their following appointment due to the backup.

Aim to review if the patient is taking each medication at the dosage and intervals they are prescribed. This is a typical task in primary care and should be done by moving down the medication list one by one. This must happen before any medication is dispensed. Outside of these directions, you will respond to the best of your knowledge. You may use what has been learned in past scenarios here.

Script

Student Role-player - “*Good morning! Sorry to keep you waiting, I am [student name here], the third-year medical student assisting with your care. Today we will be doing a medication review to make sure our records match what you are taking every day*”.

Read the *medication list:*

*Acetaminophen 325mg - active*

*Omeprazole 20mg before breakfast, lunch, and dinner -active*

*Atorvastatin 40mg daily – active/suspended*

*Lisinopril 20mg daily – inactive*

*Ibuprofen 400mg every 6 hours as needed – active*

*Hydroxychloroquine 200mg twice daily – inactive*

*Prednisone 10mg daily - inactive*

*Oxycodone 5mg every 4 hours – inactive*

At this point, please respond to the patient with the best of your knowledge. You may use what has been learned from the previous scenarios here.

**Scenario 3**

**Role - Patient Role-player**

Scene - A long day in the clinic has gone longer than expected and a patient has been left waiting in a clinic room for over an hour. Due to the overload, you have been sent in to see this patient on your own while your preceptor is handling a more complicated patient. The patient is here for a medication reconciliation and refill of the medication that she takes for her SLE, which has flared up. They are now late for their following appointment due to the backup.

Note - You will interrupt the student after being informed that the day’s plan is a medication reconciliation. They will speak very loudly and in short sentences. You are here instructed to not care what the student doctor has to say outside of getting your prescription filled. The Student role-player has been told to try to decipher the patient’s story as in the last scenario. Please interrupt the Student role-player at least five times mid-sentence. **You may construct your own angry sentences to extend the encounter or signal to end the encounter with the final line below.**

Script

**Student Role-player** - “*Good morning! Sorry to keep you waiting, I am [student name here], the third-year medical student assisting with your care. Today we will be doing a medication review to make sure our records match what you are taking every day*”

**Patient Role-player** - **“***Don't touch me. We don’t need to have a conversation. I just need my medication so I can get back to work.***”**

Other argumentative lines that can be used to interrupt the student doctor:

- “Do you know how long I’ve been sitting here? I came in an hour EARLY to try to get things moving, and this is the thanks I get? Where is my refill?”
- “*I had to take time off of WORK to be here and now you all have made me run through my lunch hour! When am I supposed to eat, DOCTOR?*”
- “*Oh you don’t want to help me! You just want me to pay my money! Well, I refuse until I get my refill!*”
- “*I’ve been here since 10 am, I really am just another number aren’t I? Well, this number isn’t leaving without a medication refill!*”
- “*My skin HURTS. My knees HURT. My hands HURT. I JUST want my medication and to get back to WORK.*”

To signal the end of the scenario, you may end with ***the final line*** - “*I’m sorry, WHO are you?? A STUDENT? Get me a real doctor in here!!”*
