# Supplementary material for: Coachability: A Longitudinal Curriculum to Promote Medical Students’ Growth Mindset, Feedback Utilization, and Resilience
Source: MedEdPORTAL. 2024 Oct 11;20:11450. doi: 10.15766/mep_2374-8265.11450 (PMC11467082; doi:10.15766/mep_2374-8265.11450)
Supplement: Supplementary file 1 — Year 1 - Coachability.pptxYear 1 - Self-Assessment.docxYear 2 - Coachability.pptxSeminar 1 - Facilitator Guide.docxSeminar 2 - Facilitator Guide.docxSeminar 3 - Facilitator Guide.docxPostseminar Survey.docxFocus Group Protocol.docx [file mep_2374-8265.11450-s001.zip › G. Postseminar Survey.docx]

**Coachability Curriculum Evaluation**

*Post-Intervention Survey – Year 2 Seminars*

Please circle today’s seminar:

**Feedback** **Conflict Resolution/Resilience** **Coachability from Patients**

- **What is one lesson that you will take away from today’s seminar?**
- **How will you apply this lesson in the future?**
- **How could this seminar be improved?**

For the following questions, please circle your response:

- The material discussed in today’s seminar is important.

**Strongly Agree Agree Neutral Disagree Strongly Disagree**

- Today’s seminar was a valuable learning experience.

**Strongly Agree Agree Neutral Disagree Strongly Disagree**

- Today’s seminar was engaging.

**Strongly Agree Agree Neutral Disagree Strongly Disagree**

- I would recommend today’s seminar to my peers.

**Strongly Agree Agree Neutral Disagree Strongly Disagree**
