# Supplementary material for: Coachability: A Longitudinal Curriculum to Promote Medical Students’ Growth Mindset, Feedback Utilization, and Resilience
Source: MedEdPORTAL. 2024 Oct 11;20:11450. doi: 10.15766/mep_2374-8265.11450 (PMC11467082; doi:10.15766/mep_2374-8265.11450)
Supplement: Supplementary file 1 — Year 1 - Coachability.pptxYear 1 - Self-Assessment.docxYear 2 - Coachability.pptxSeminar 1 - Facilitator Guide.docxSeminar 2 - Facilitator Guide.docxSeminar 3 - Facilitator Guide.docxPostseminar Survey.docxFocus Group Protocol.docx [file mep_2374-8265.11450-s001.zip › H. Focus Group Protocol.docx]

**Coachability Curriculum Evaluation**

*Focus Group Protocol - Year 2 Seminars*

Purpose: This protocol is for exploring medical students’ experiences in the coachability curriculum Year 2 seminars.

Scope of use: Medical students who have completed the Coachability Curriculum and have completed at least one clinical clerkship

Introductory remarks:

Review the Coachability Curriculum, highlighting the key topics and activities of each Year 2 Seminar:

- Session 1 - Feedback
  - One student drawing and receiving feedback, one student judging and giving feedback
- Session 2 - Conflict Resolution
  - Discussion of common conflicts encountered during second year and the steps to effective conflict resolution
- Session 3 - Resilience
  - Modified Maslach burnout inventory
  - Discussion of common reasons people experience burnout

Focus group questions:

- What was your favorite part of the coachability small-group sessions? Why?
- What was your least favorite part of the coachability small-group sessions? Why?
- How do you feel the coachability small-group sessions helped you throughout clerkships?
- Were there strategies/concepts discussed during the Coachability curriculum that you feel were not effective during your clerkships?
- Have you had difficulty receiving effective feedback during clerkships? If so, how did you deal with that after completing the coachability curriculum?
- Have you experienced interpersonal conflict during clerkships (with a peer, resident, attending, etc.)? If so, how did you deal with that after completing the coachability curriculum?
- Have you or are you currently experiencing burnout? If so, how did/are you dealing with that after completing the coachability curriculum?
- Is there anything else you would like to mention about the Coachability curriculum that we haven’t discussed?
