# Supplementary material for: Coachability: A Longitudinal Curriculum to Promote Medical Students’ Growth Mindset, Feedback Utilization, and Resilience
Source: MedEdPORTAL. 2024 Oct 11;20:11450. doi: 10.15766/mep_2374-8265.11450 (PMC11467082; doi:10.15766/mep_2374-8265.11450)
Supplement: Supplementary file 1 — Year 1 - Coachability.pptxYear 1 - Self-Assessment.docxYear 2 - Coachability.pptxSeminar 1 - Facilitator Guide.docxSeminar 2 - Facilitator Guide.docxSeminar 3 - Facilitator Guide.docxPostseminar Survey.docxFocus Group Protocol.docx [file mep_2374-8265.11450-s001.zip › A. Year 1 - Coachability.pptx]

## Slide 1
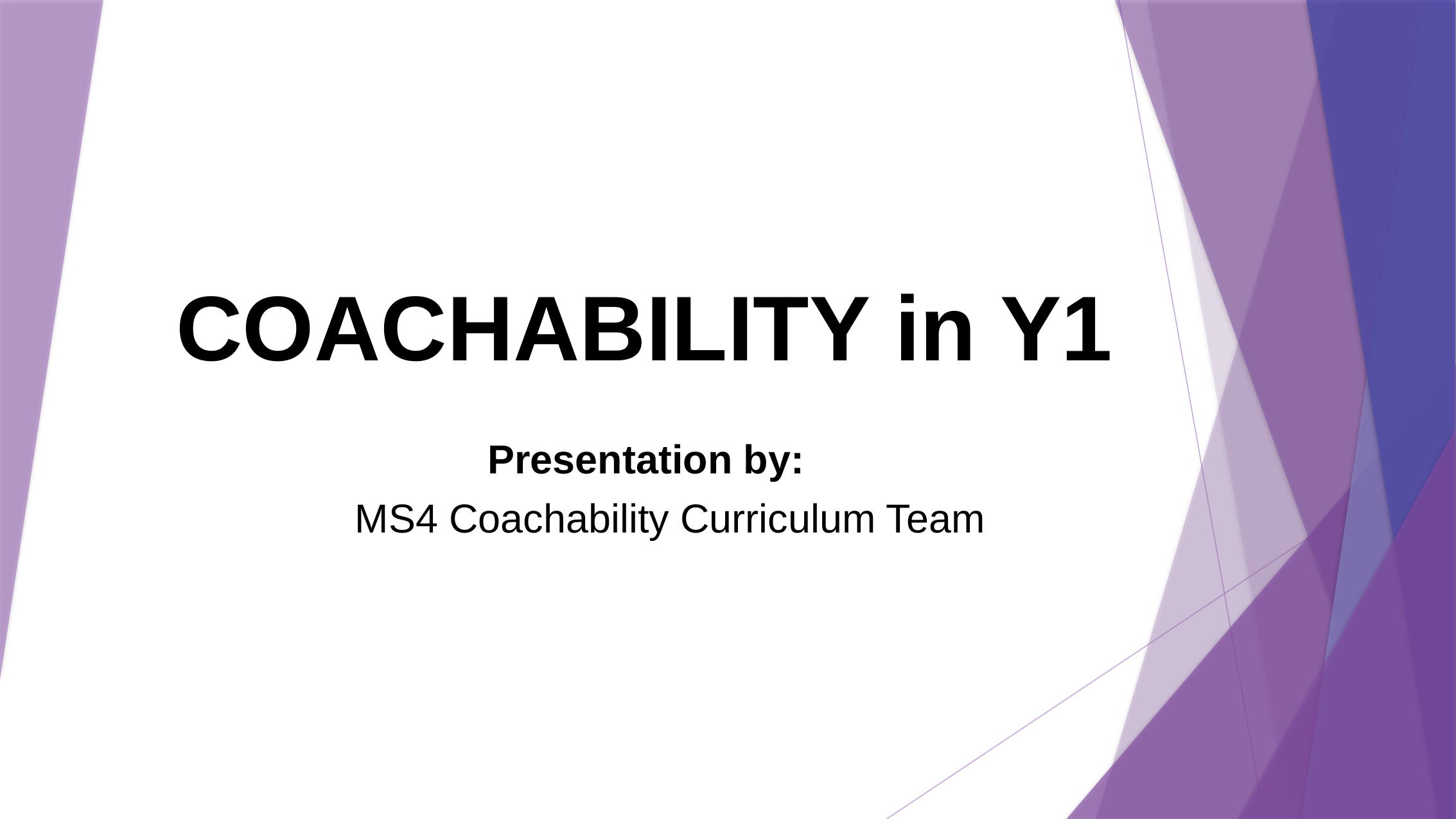

# COACHABILITY in Y1
Presentation by:
MS4 Coachability Curriculum Team

## Slide 2
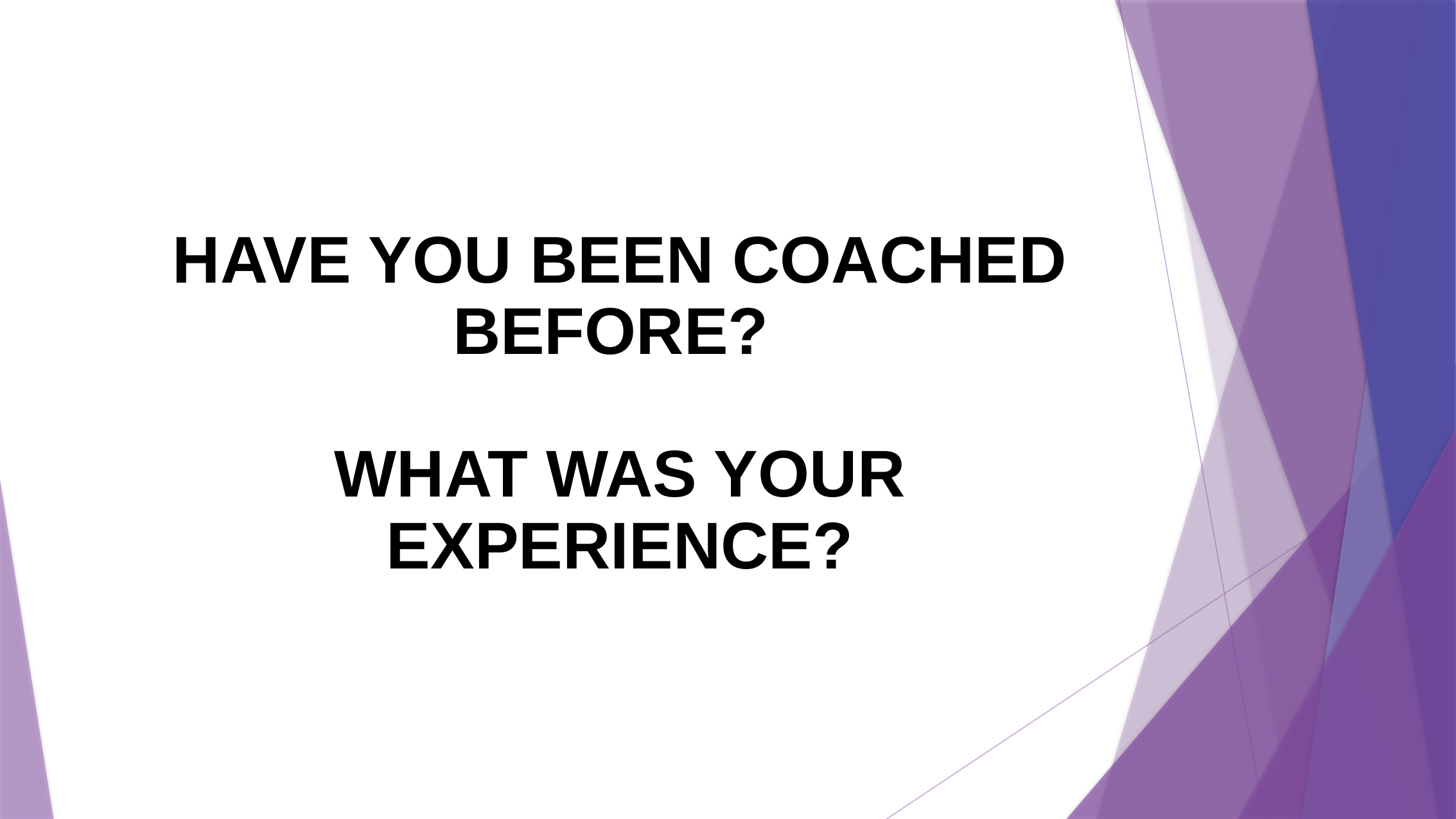

HAVE YOU BEEN COACHED BEFORE?
WHAT WAS YOUR EXPERIENCE?

## Slide 3
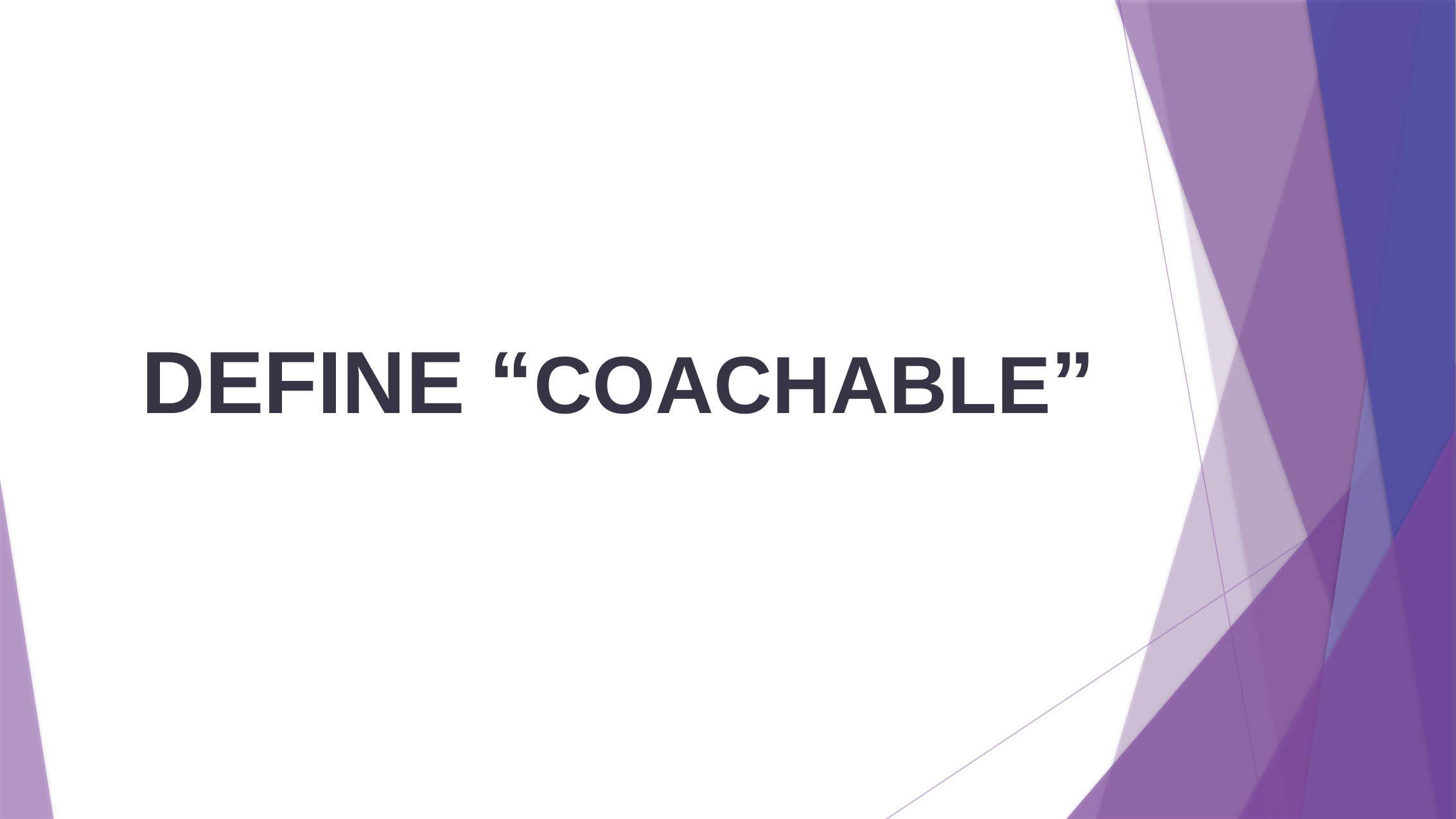

# DEFINE “COACHABLE”

## Slide 4
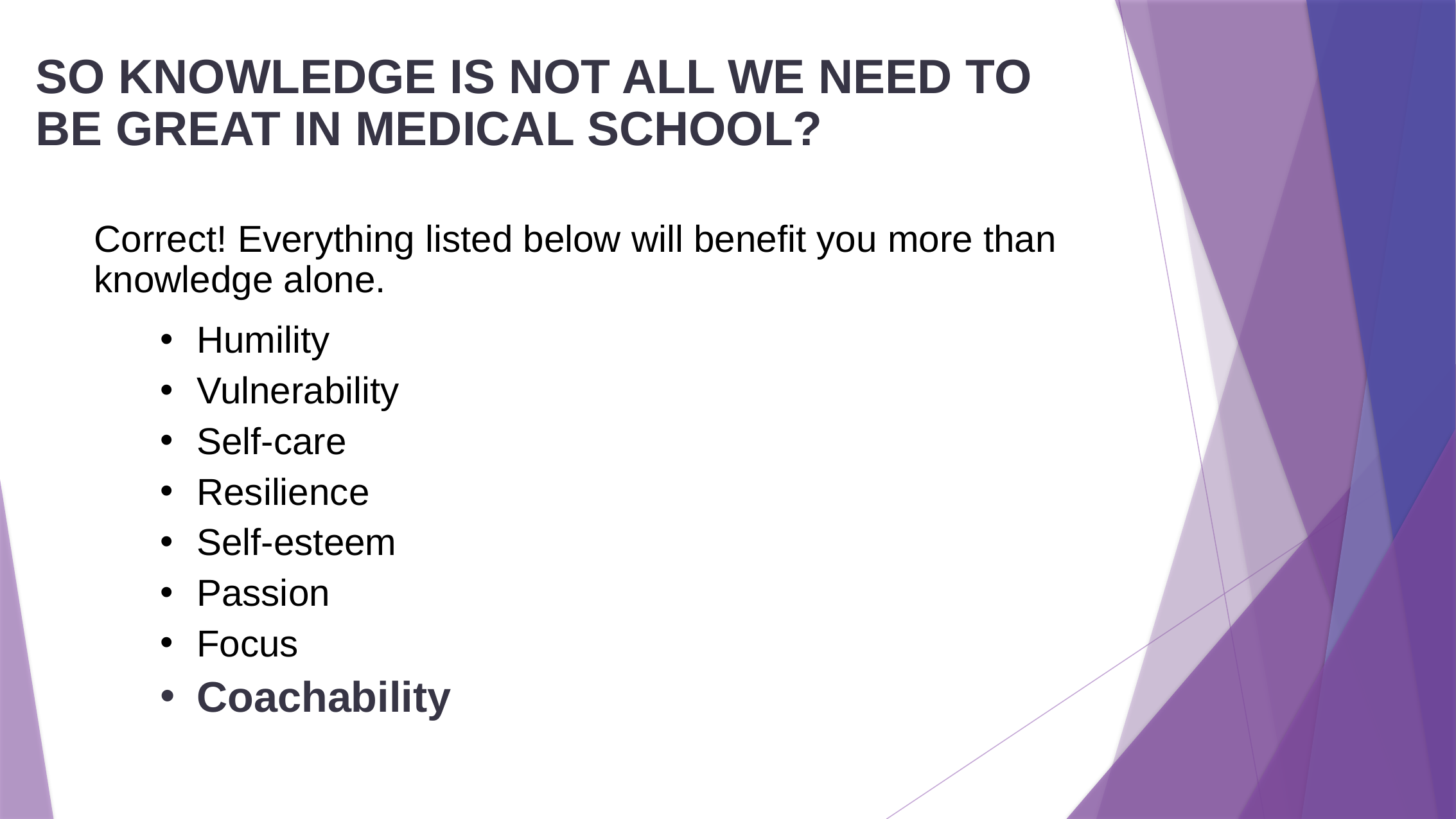

# SO KNOWLEDGE IS NOT ALL WE NEED TO BE GREAT IN MEDICAL SCHOOL?
Correct! Everything listed below will benefit you more than knowledge alone.
Humility
Vulnerability
Self-care
Resilience
Self-esteem
Passion
Focus
Coachability

## Slide 5
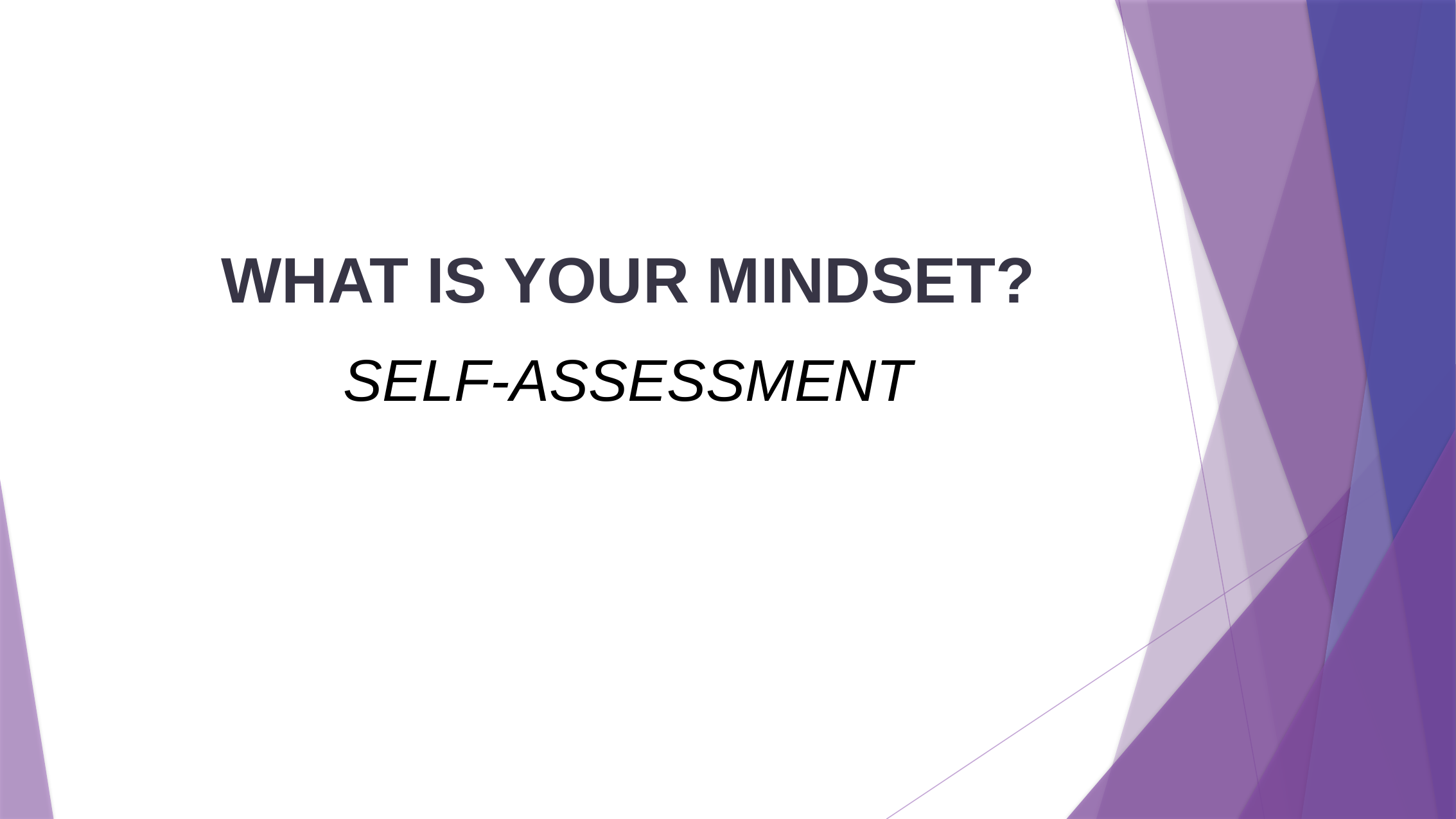

WHAT IS YOUR MINDSET?
SELF-ASSESSMENT

## Slide 6
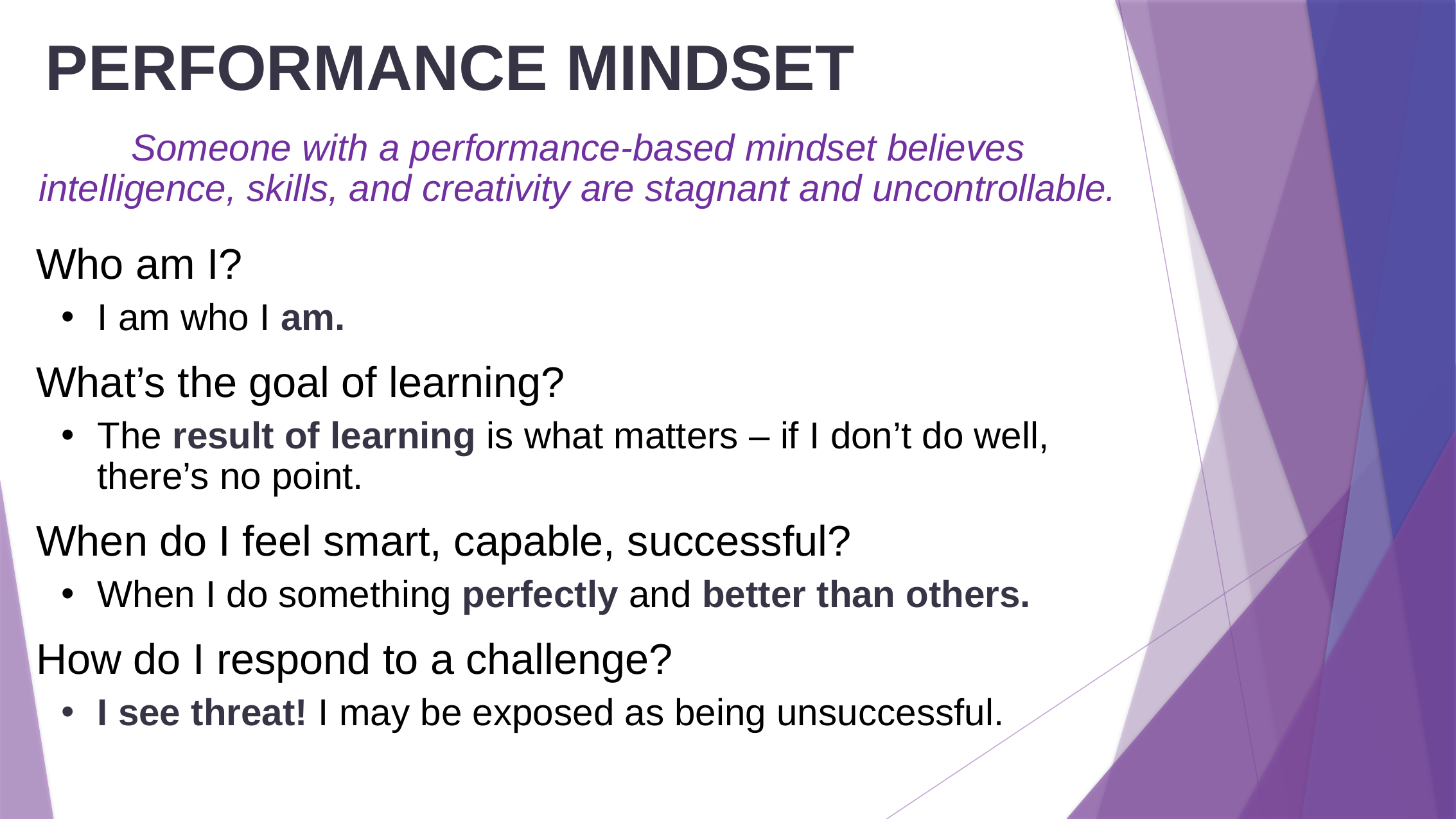

# PERFORMANCE MINDSET
Someone with a performance-based mindset believes intelligence, skills, and creativity are stagnant and uncontrollable.
Who am I?
I am who I am.
What’s the goal of learning?
The result of learning is what matters – if I don’t do well, there’s no point.
When do I feel smart, capable, successful?
When I do something perfectly and better than others.
How do I respond to a challenge?
I see threat! I may be exposed as being unsuccessful.

## Slide 7
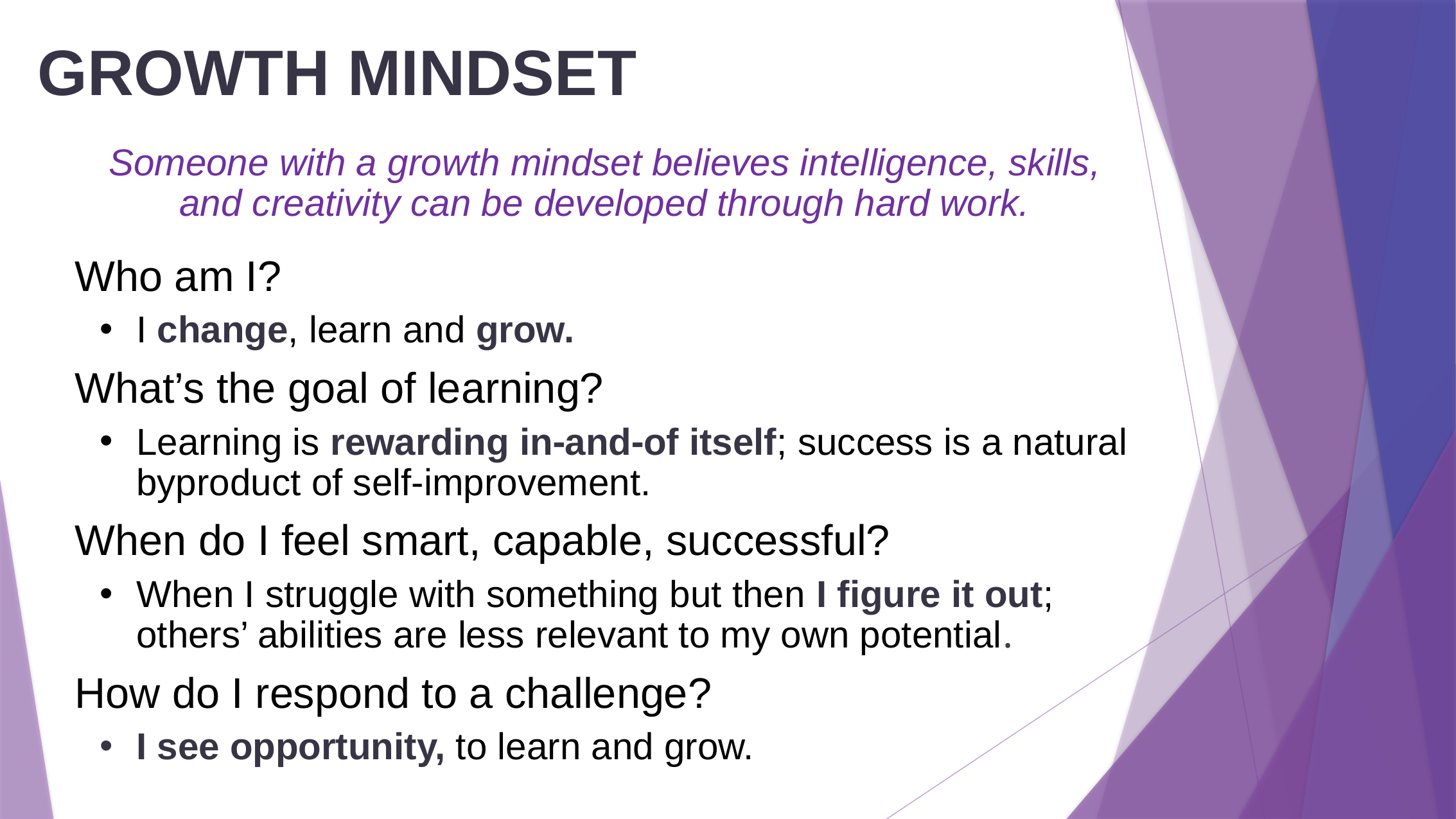

# GROWTH MINDSET
Someone with a growth mindset believes intelligence, skills, and creativity can be developed through hard work.
Who am I?
I change, learn and grow.
What’s the goal of learning?
Learning is rewarding in-and-of itself; success is a natural byproduct of self-improvement.
When do I feel smart, capable, successful?
When I struggle with something but then I figure it out; others’ abilities are less relevant to my own potential.
How do I respond to a challenge?
I see opportunity, to learn and grow.

## Slide 8
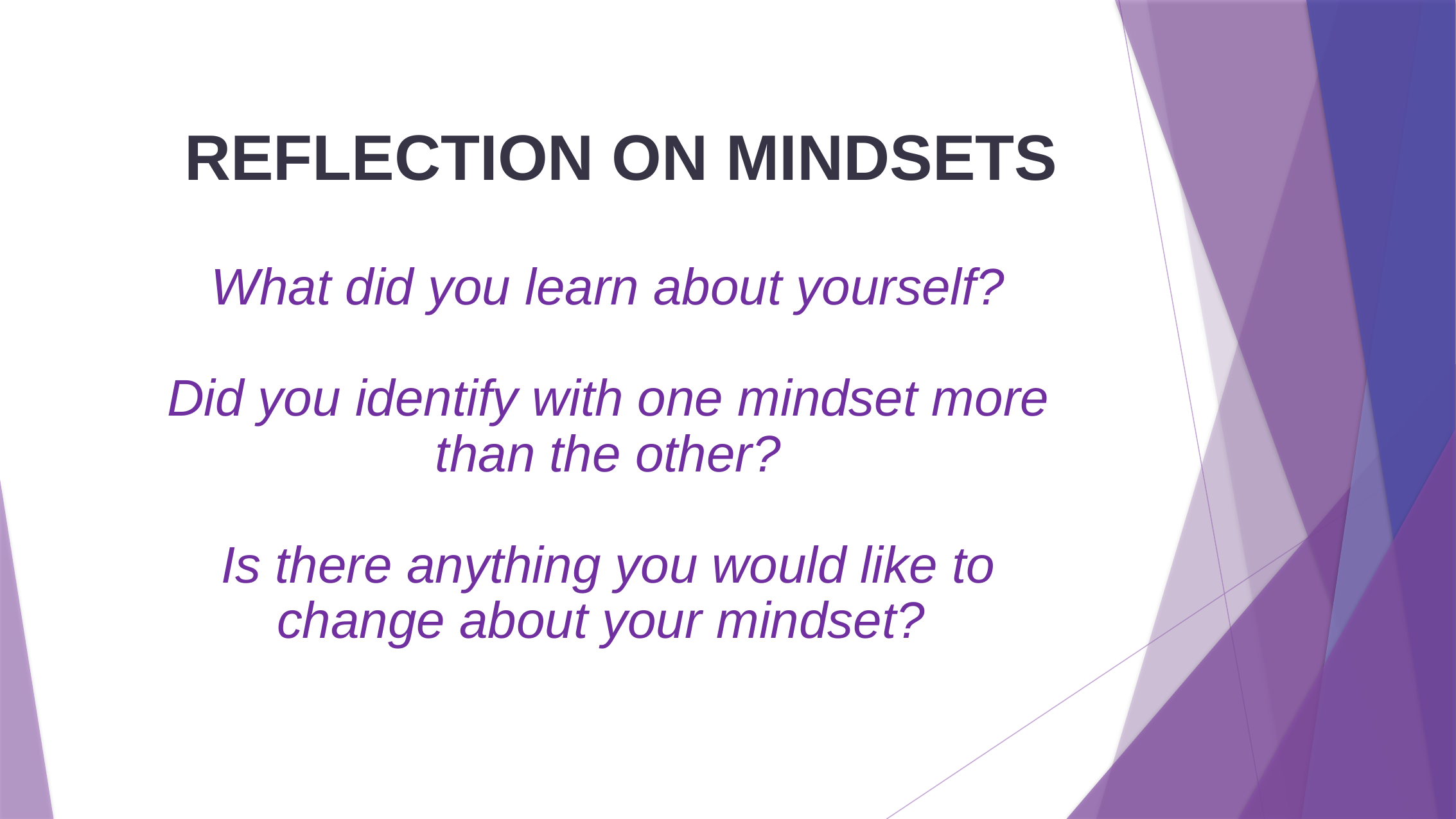

# REFLECTION ON MINDSETS
What did you learn about yourself?
Did you identify with one mindset more than the other?
Is there anything you would like to change about your mindset?

## Slide 9
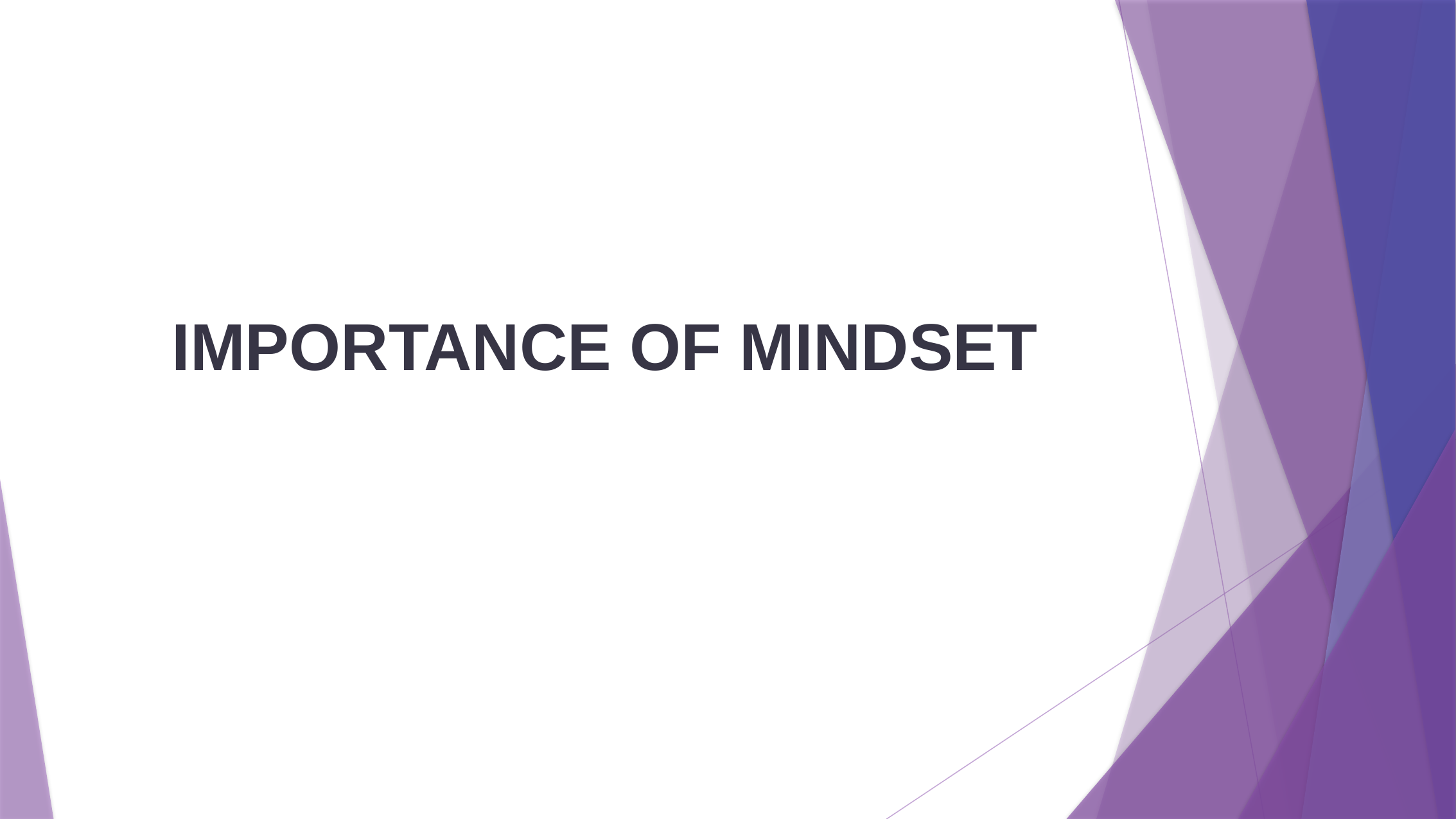

# IMPORTANCE OF MINDSET

## Slide 10
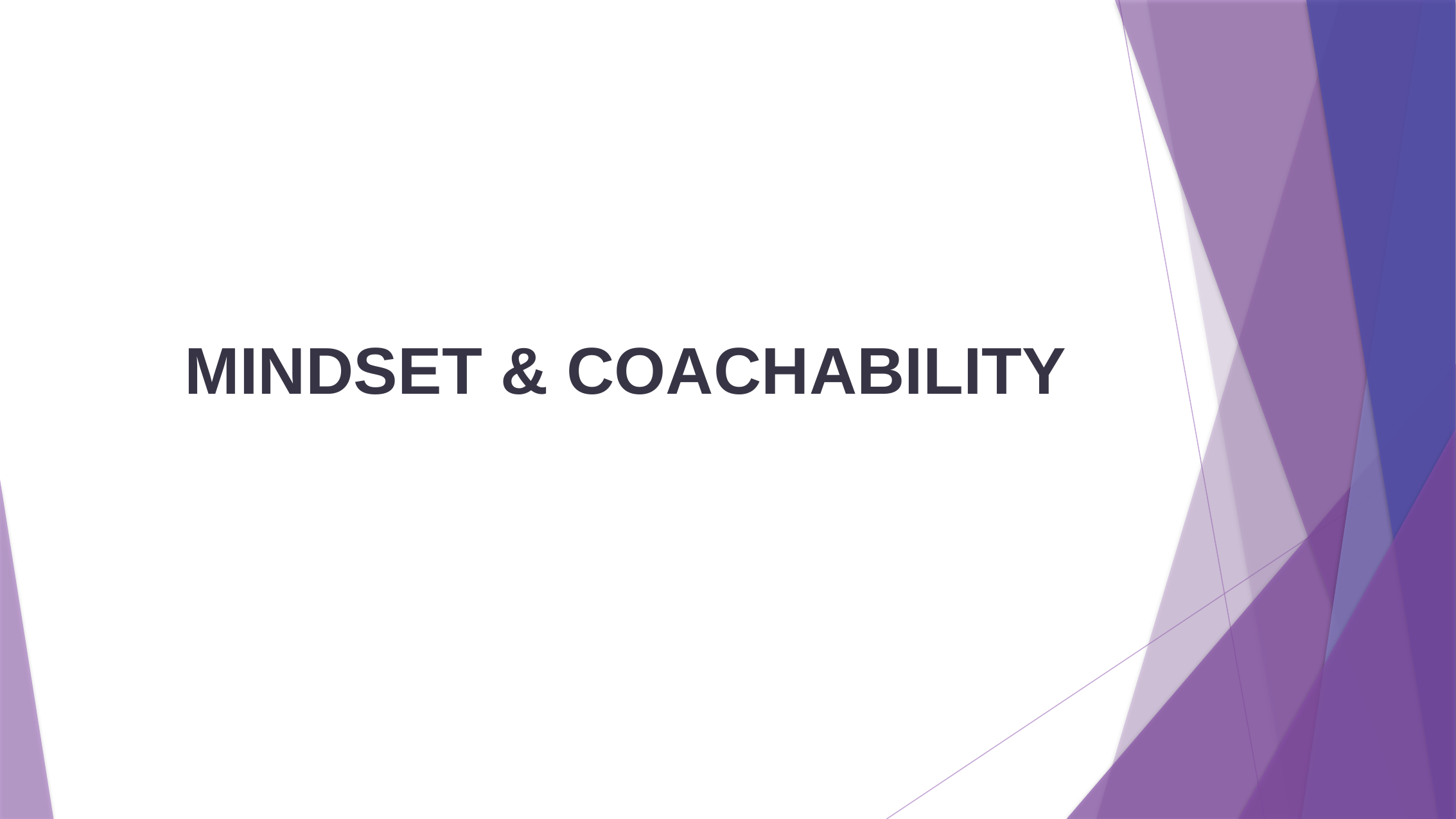

# MINDSET & COACHABILITY

## Slide 11
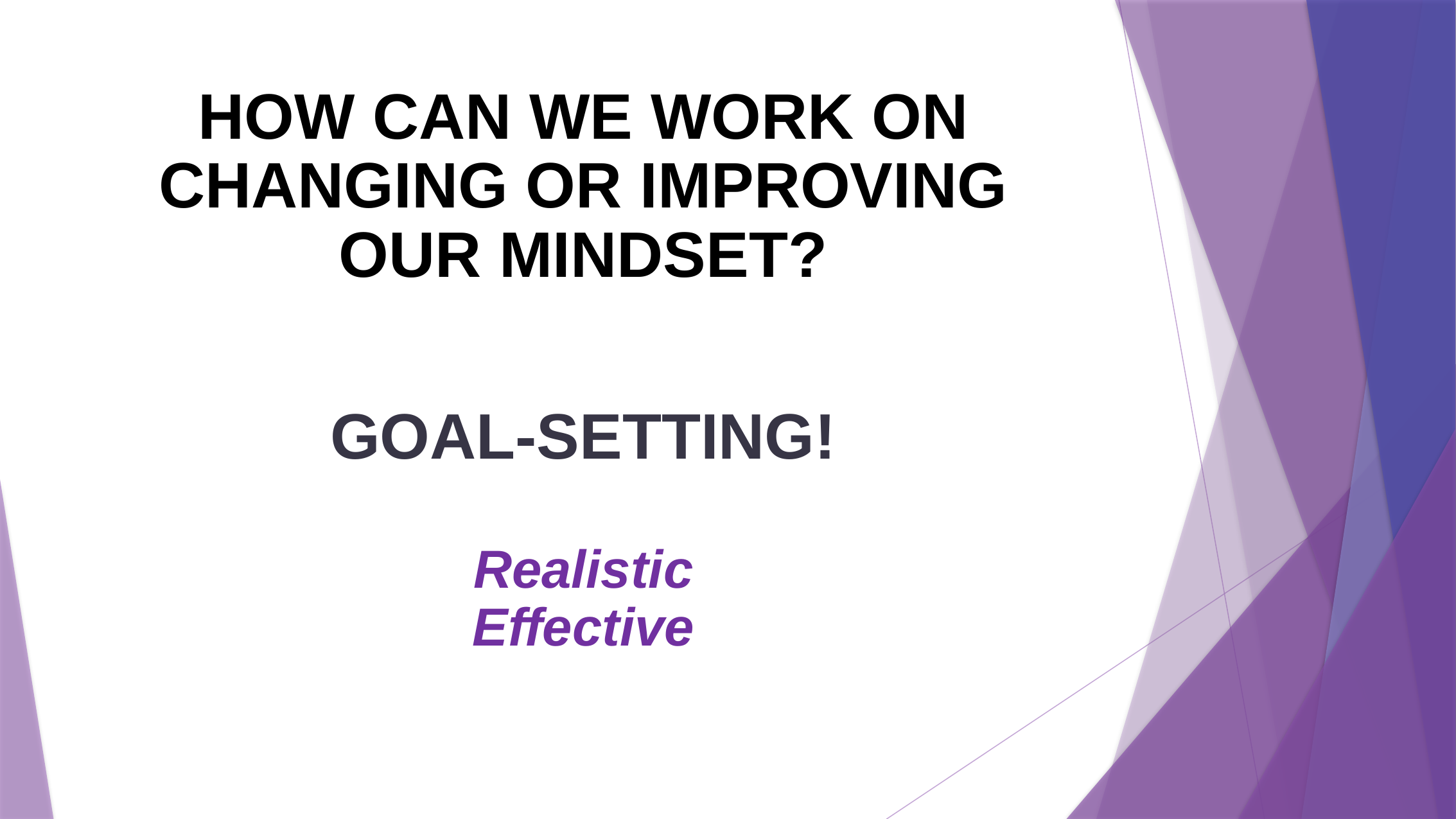

# HOW CAN WE WORK ON CHANGING OR IMPROVING OUR MINDSET?
GOAL-SETTING!
Realistic
Effective

## Slide 12
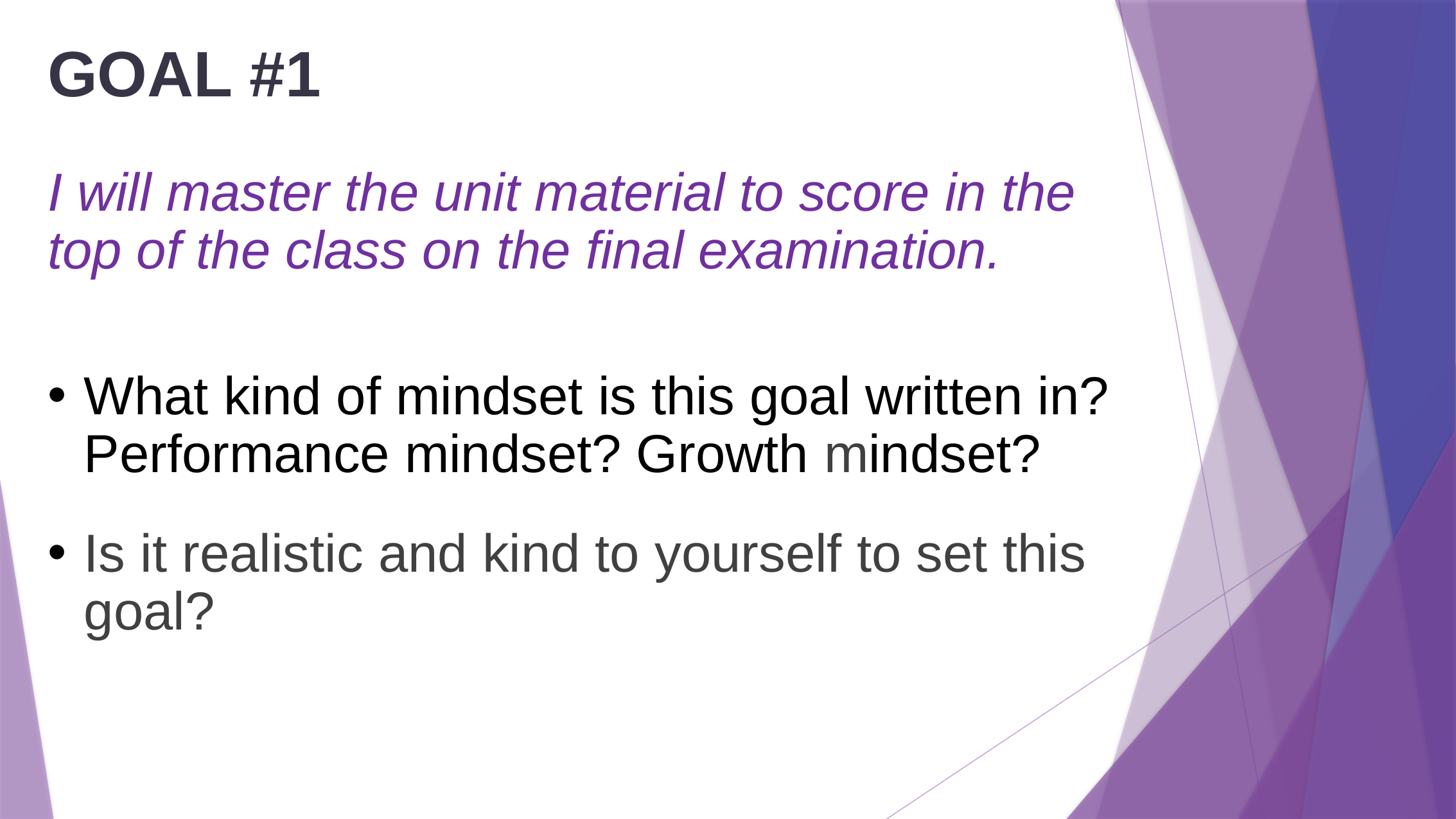

# GOAL #1
I will master the unit material to score in the top of the class on the final examination.
What kind of mindset is this goal written in? Performance mindset? Growth mindset?
Is it realistic and kind to yourself to set this goal?

## Slide 13
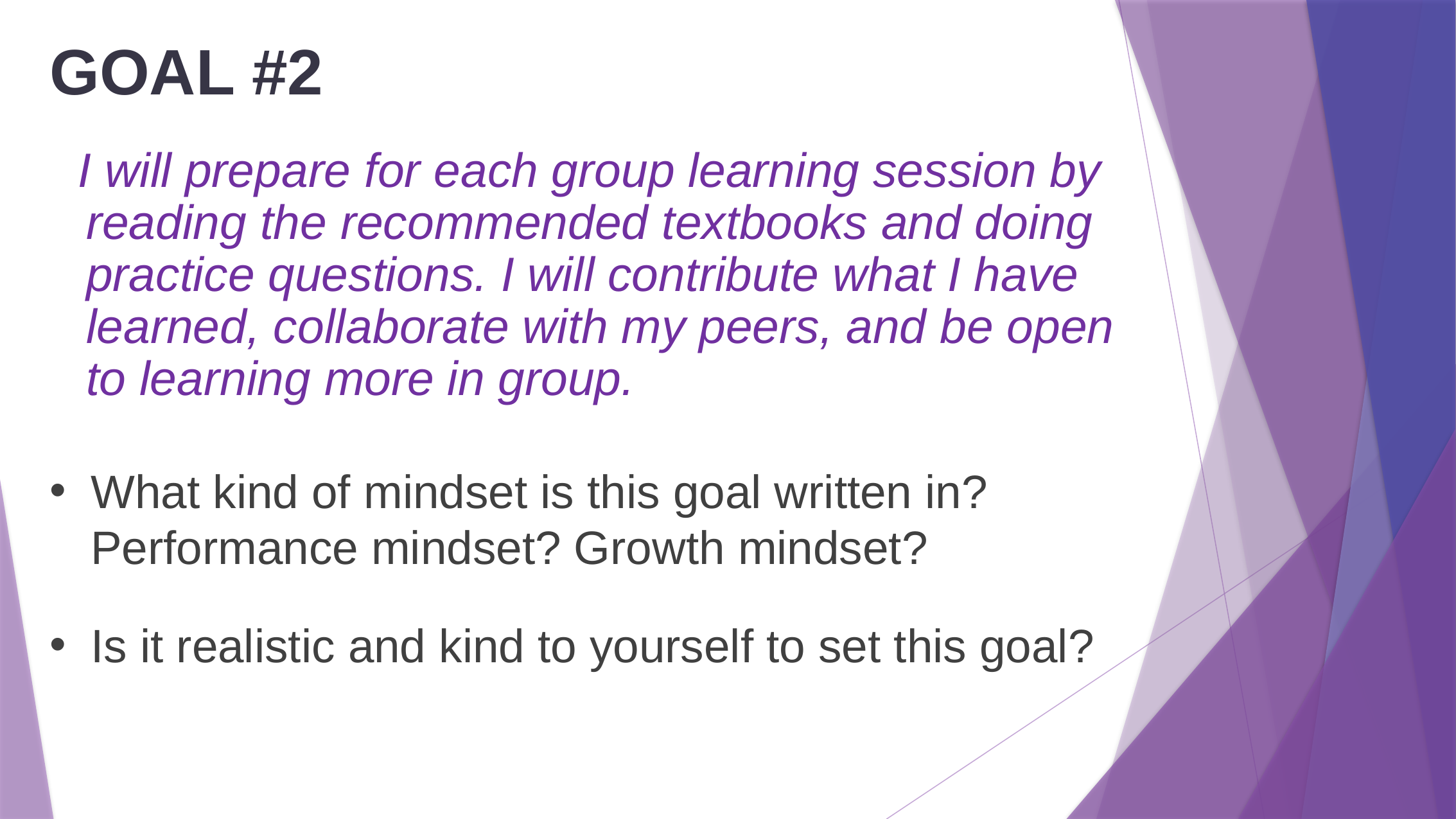

# GOAL #2
I will prepare for each group learning session by reading the recommended textbooks and doing practice questions. I will contribute what I have learned, collaborate with my peers, and be open to learning more in group.
What kind of mindset is this goal written in? Performance mindset? Growth mindset?
Is it realistic and kind to yourself to set this goal?

## Slide 14
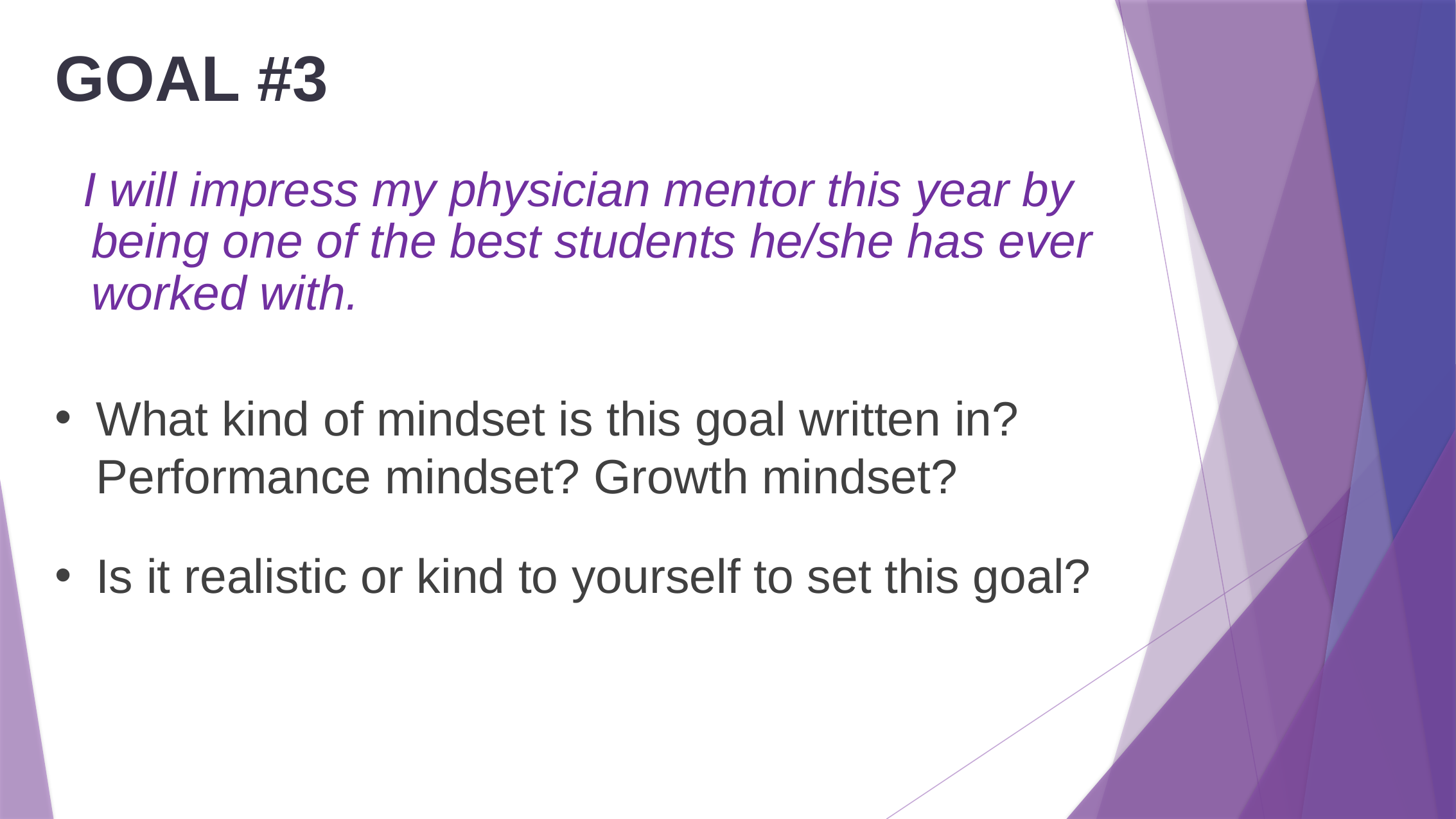

# GOAL #3
I will impress my physician mentor this year by being one of the best students he/she has ever worked with.
What kind of mindset is this goal written in? Performance mindset? Growth mindset?
Is it realistic or kind to yourself to set this goal?

## Slide 15
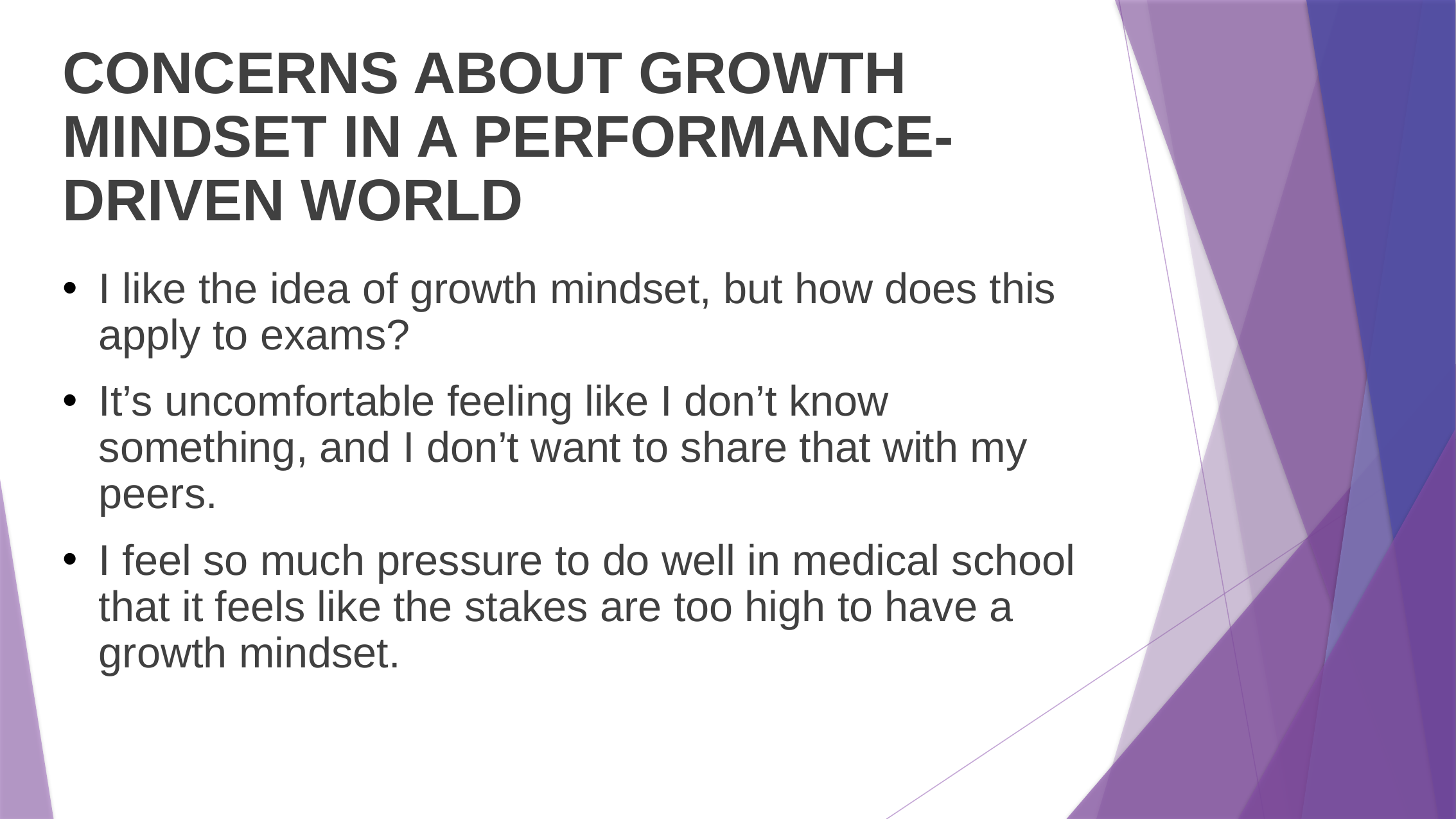

# CONCERNS ABOUT GROWTH MINDSET IN A PERFORMANCE-DRIVEN WORLD
I like the idea of growth mindset, but how does this apply to exams?
It’s uncomfortable feeling like I don’t know something, and I don’t want to share that with my peers.
I feel so much pressure to do well in medical school that it feels like the stakes are too high to have a growth mindset.

## Slide 16
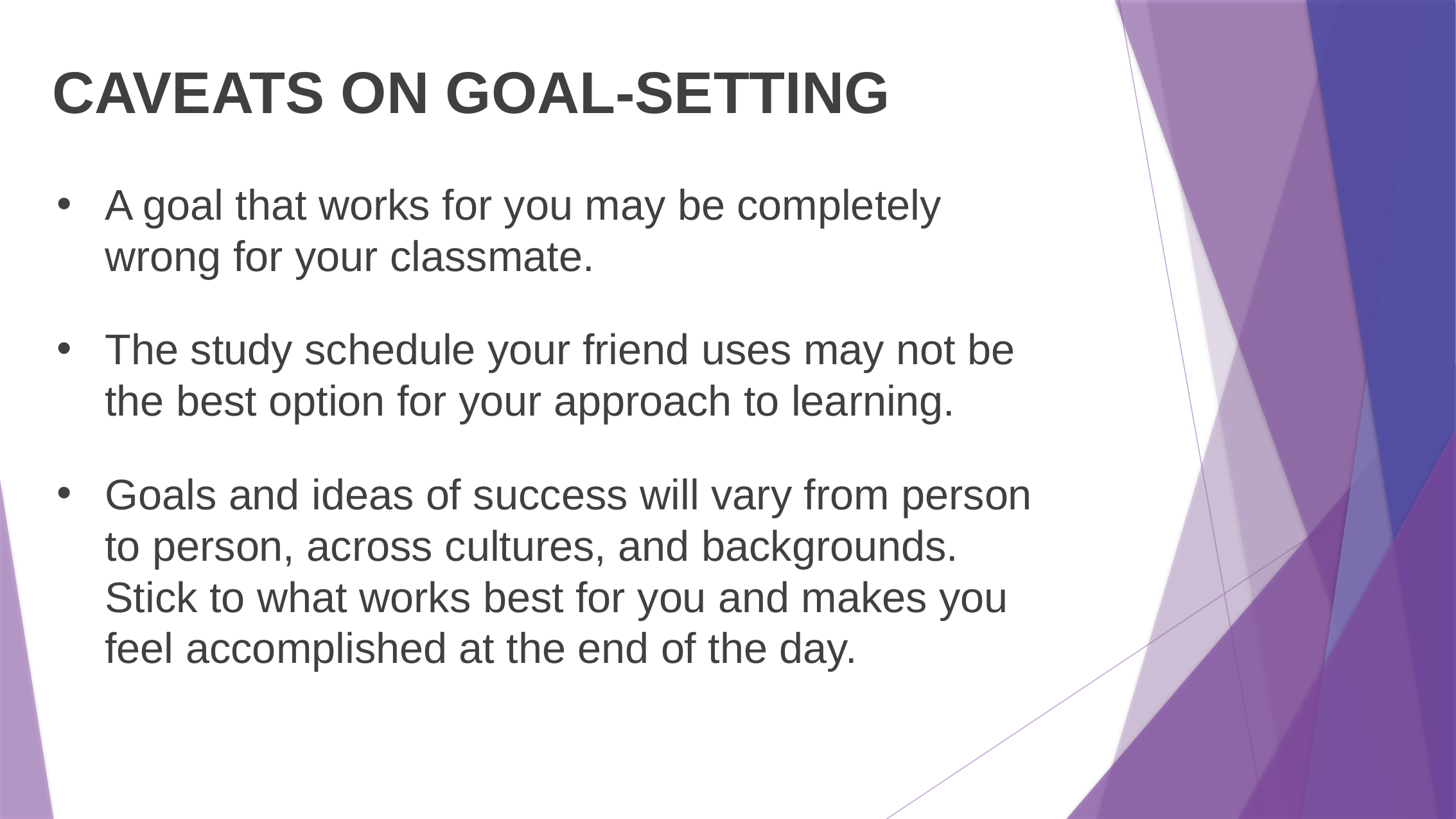

# CAVEATS ON GOAL-SETTING
A goal that works for you may be completely wrong for your classmate.
The study schedule your friend uses may not be the best option for your approach to learning.
Goals and ideas of success will vary from person to person, across cultures, and backgrounds. Stick to what works best for you and makes you feel accomplished at the end of the day.

## Slide 17
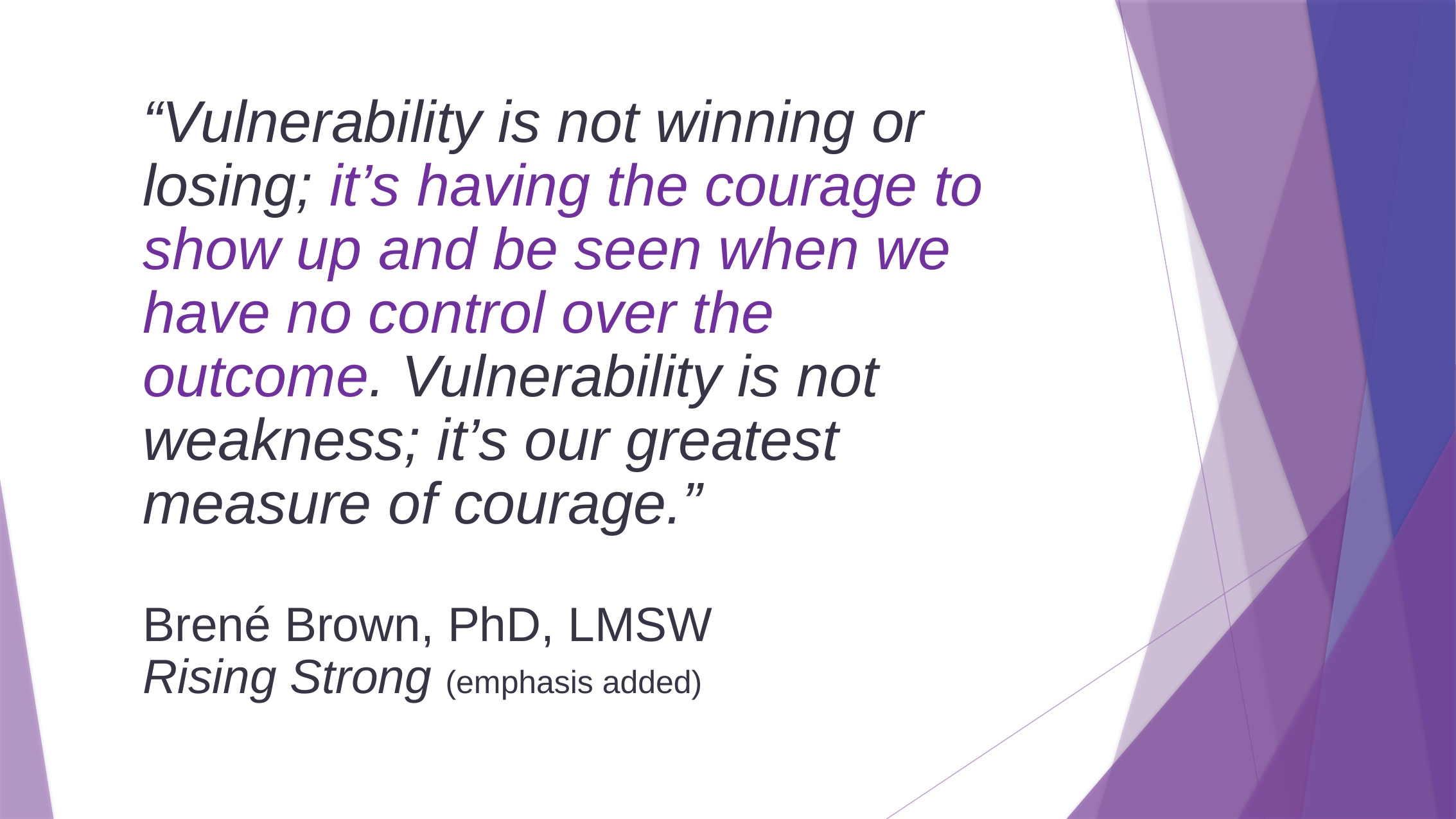

# “Vulnerability is not winning or losing; it’s having the courage to show up and be seen when we have no control over the outcome. Vulnerability is not weakness; it’s our greatest measure of courage.”
Brené Brown, PhD, LMSW
Rising Strong (emphasis added)

## Slide 18
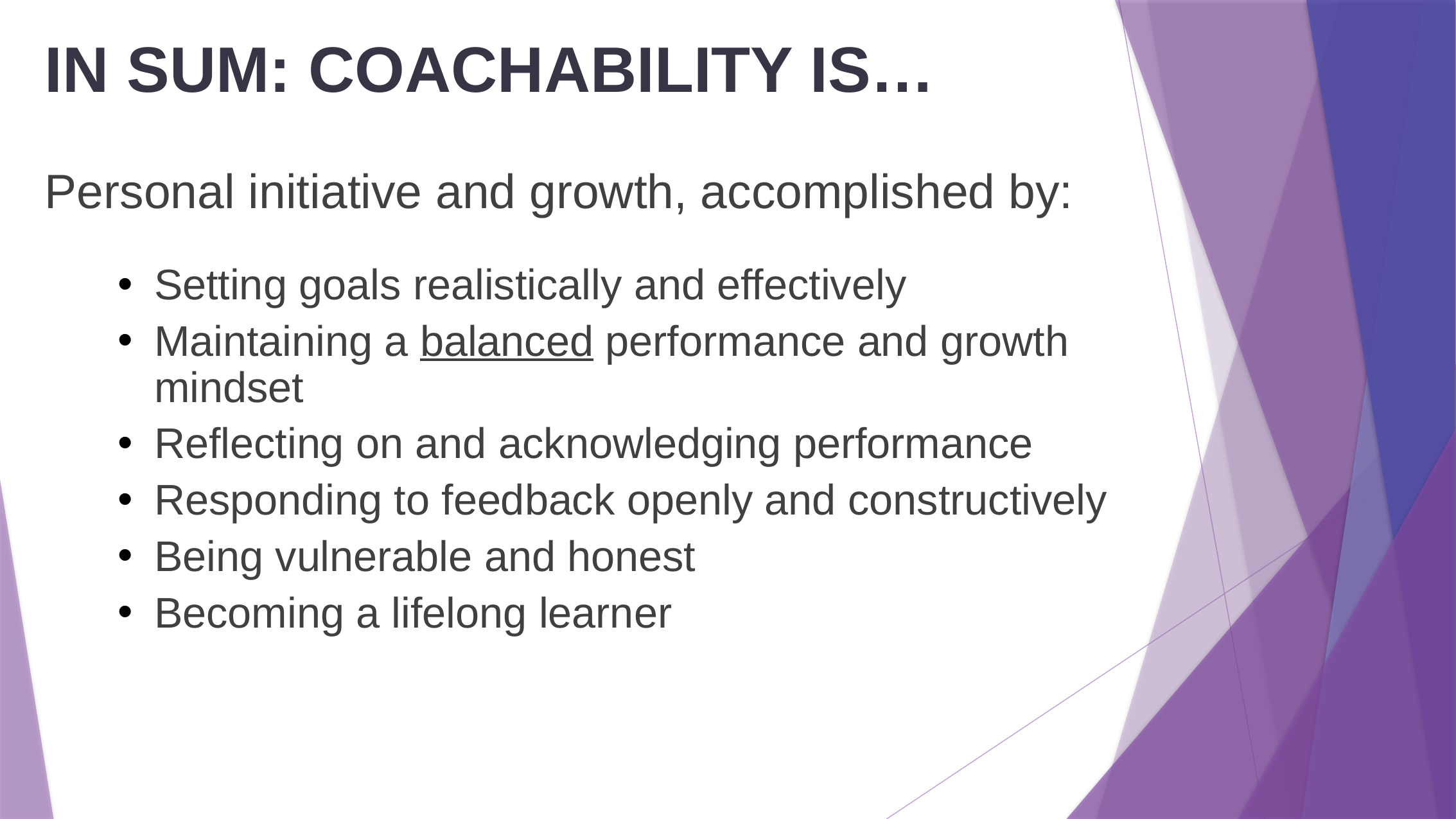

# IN SUM: COACHABILITY IS…
Personal initiative and growth, accomplished by:
Setting goals realistically and effectively
Maintaining a balanced performance and growth mindset
Reflecting on and acknowledging performance
Responding to feedback openly and constructively
Being vulnerable and honest
Becoming a lifelong learner

## Slide 19
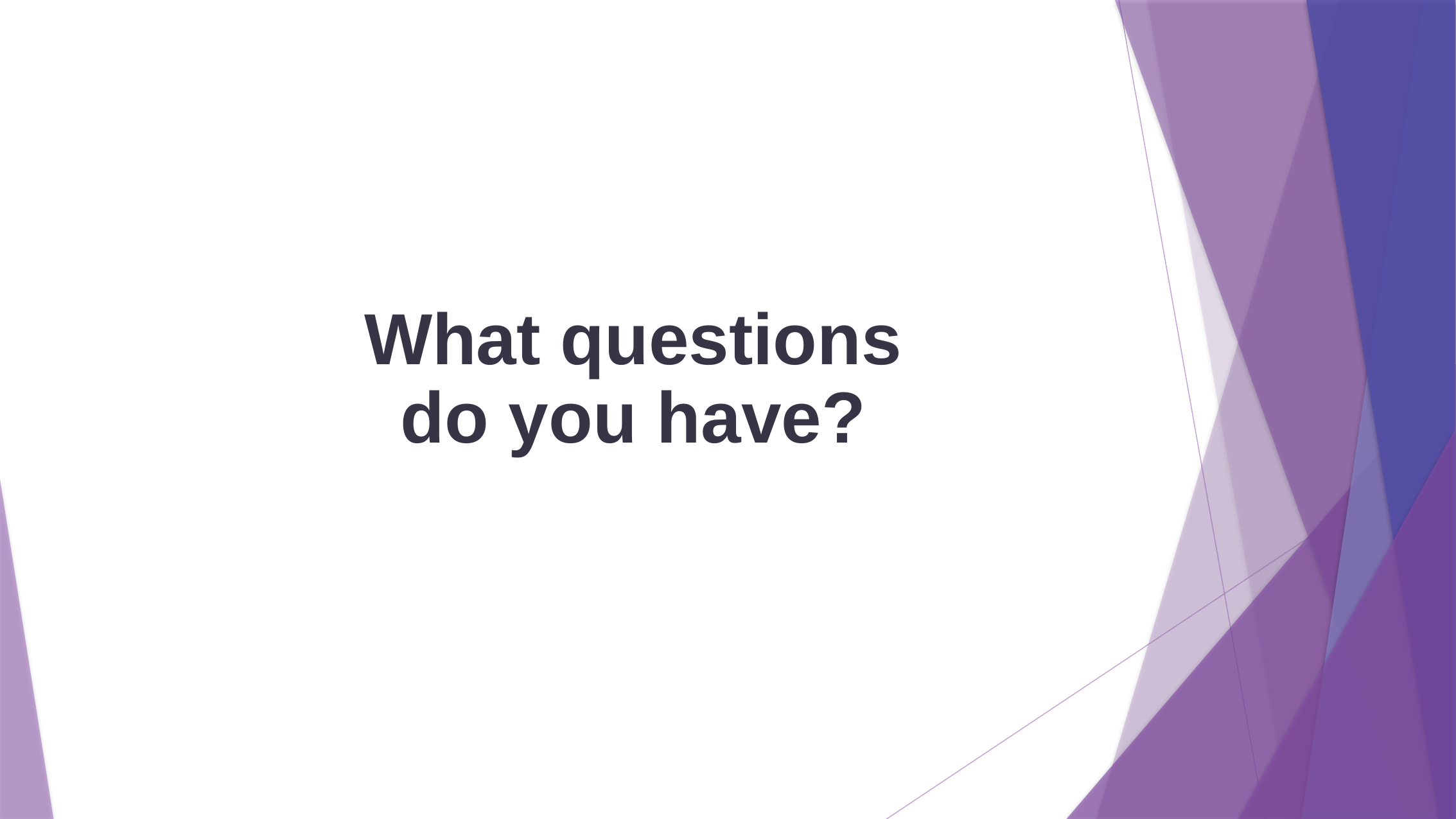

# What questions do you have?
