# Supplementary material for: Coachability: A Longitudinal Curriculum to Promote Medical Students’ Growth Mindset, Feedback Utilization, and Resilience
Source: MedEdPORTAL. 2024 Oct 11;20:11450. doi: 10.15766/mep_2374-8265.11450 (PMC11467082; doi:10.15766/mep_2374-8265.11450)
Supplement: Supplementary file 1 — Year 1 - Coachability.pptxYear 1 - Self-Assessment.docxYear 2 - Coachability.pptxSeminar 1 - Facilitator Guide.docxSeminar 2 - Facilitator Guide.docxSeminar 3 - Facilitator Guide.docxPostseminar Survey.docxFocus Group Protocol.docx [file mep_2374-8265.11450-s001.zip › B. Year 1 - Self-Assessment.docx]

**SELF-ASSESSMENT**

**What is Your Mindset?**

Please use the scale below to rate how true each of the following statements is for you.

| **1** | **2** | **3** | **4** | **5** |
| --- | --- | --- | --- | --- |
| NOT AT ALL TRUE FOR ME |  | SOMEWHAT TRUE FOR ME |  | VERY TRUE  FOR ME |

|  | **Statement** | **1** | **2** | **3** | **4** | **5** |
| --- | --- | --- | --- | --- | --- | --- |
| 1 | I enjoy studying difficult topics because I enjoy the challenge of learning new things |  |  |  |  |  |
| 2 | Truly smart people don't need to work hard or study |  |  |  |  |  |
| 3 | It’s important to me to thoroughly understand my assignments for the sake of my own learning |  |  |  |  |  |
| 4 | It is important to me to not look unintelligent in front of faculty or classmates |  |  |  |  |  |
| 5 | I see mistakes as something to learn from rather than failures |  |  |  |  |  |
| 6 | I’d rather wait to do something until I’m confident it can be done well. |  |  |  |  |  |
| 7 | The harder you work at something, the more you will improve |  |  |  |  |  |
| 8 | I often feel hurt or upset when receiving feedback about my performance |  |  |  |  |  |
| 9 | Improvement is more important than perfection |  |  |  |  |  |
| 10 | Grades are the most important indicator of my success |  |  |  |  |  |

Total and interpret your results on the next page.

Record your ratings for each statement in the grid below:

| **Growth Mindset** | | **Performance Mindset** | |
| --- | --- | --- | --- |
| **1** |  | **2** |  |
| **3** |  | **4** |  |
| **5** |  | **6** |  |
| **7** |  | **8** |  |
| **9** |  | **10** |  |
| **TOTAL** |  | **TOTAL** |  |

**Key for GROWTH Mindset**

| **20-25** | You focus strongly on growth & lifelong learning |
| --- | --- |
| **14-19** | You value personal growth greater than or equal to impressing others or performing well |
| **5-13** | You more often place importance on performance over personal growth |

**Key for PERFORMANCE Mindset**

| **20-25** | You focus strongly on performance & seeking perfection |
| --- | --- |
| **14-19** | You value grades and evaluations greater than or equal to personal growth |
| **5-13** | You more often place importance on personal growth over performance |

Adapted from Dweck, C.S. (2006) Mindset: The new psychology of success. New York House Inc.
