# Supplementary material for: Coachability: A Longitudinal Curriculum to Promote Medical Students’ Growth Mindset, Feedback Utilization, and Resilience
Source: MedEdPORTAL. 2024 Oct 11;20:11450. doi: 10.15766/mep_2374-8265.11450 (PMC11467082; doi:10.15766/mep_2374-8265.11450)
Supplement: Supplementary file 1 — Year 1 - Coachability.pptxYear 1 - Self-Assessment.docxYear 2 - Coachability.pptxSeminar 1 - Facilitator Guide.docxSeminar 2 - Facilitator Guide.docxSeminar 3 - Facilitator Guide.docxPostseminar Survey.docxFocus Group Protocol.docx [file mep_2374-8265.11450-s001.zip › C. Year 2 - Coachability.pptx]

## Slide 1
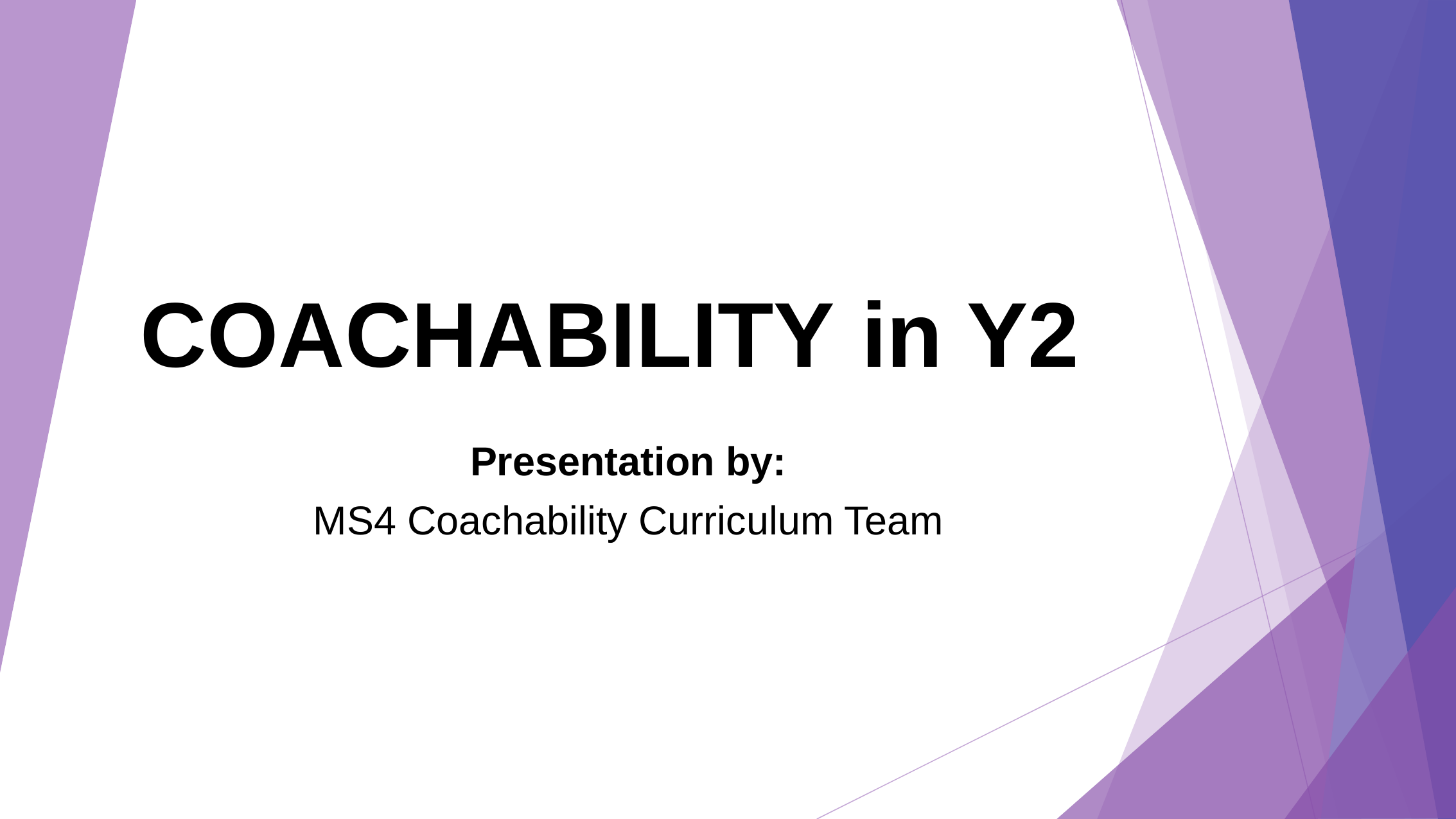

COACHABILITY in Y2
Presentation by:
MS4 Coachability Curriculum Team

## Slide 2
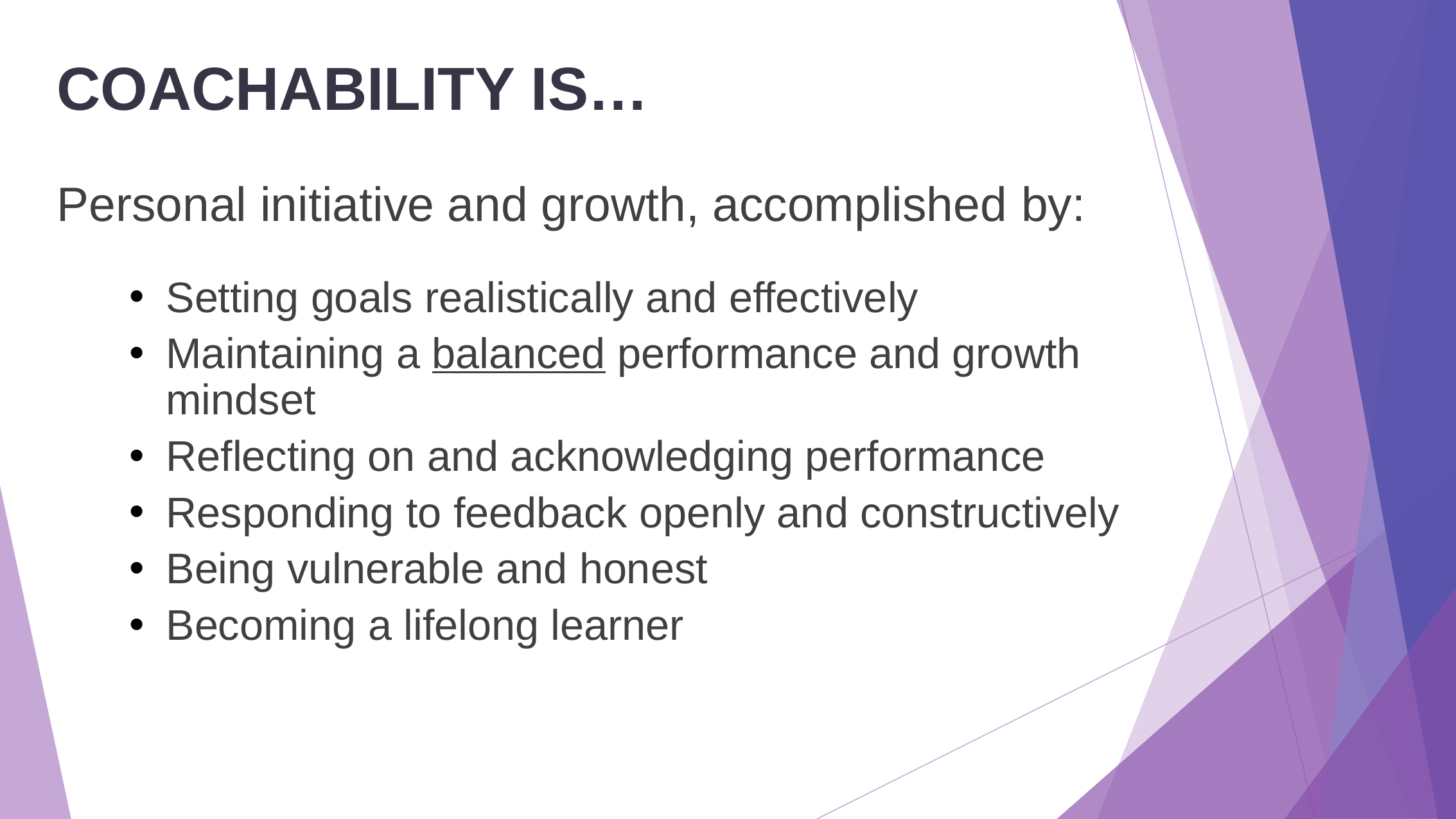

# COACHABILITY IS…
Personal initiative and growth, accomplished by:
Setting goals realistically and effectively
Maintaining a balanced performance and growth mindset
Reflecting on and acknowledging performance
Responding to feedback openly and constructively
Being vulnerable and honest
Becoming a lifelong learner

## Slide 3
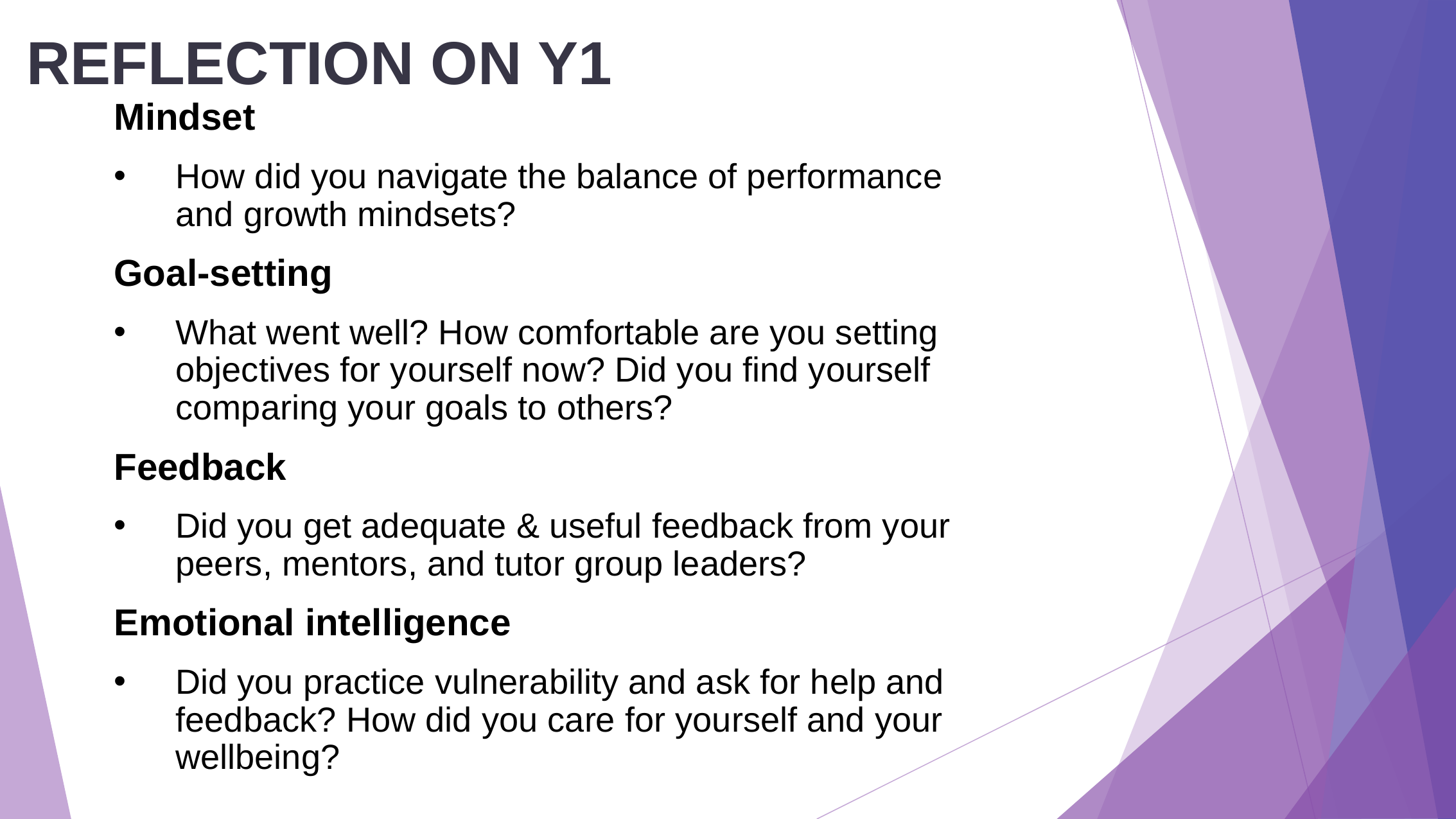

# Y1 COACHABILITY RECAP
REFLECTION ON Y1
Mindset
How did you navigate the balance of performance and growth mindsets?
Goal-setting
What went well? How comfortable are you setting objectives for yourself now? Did you find yourself comparing your goals to others?
Feedback
Did you get adequate & useful feedback from your peers, mentors, and tutor group leaders?
Emotional intelligence
Did you practice vulnerability and ask for help and feedback? How did you care for yourself and your wellbeing?

## Slide 4
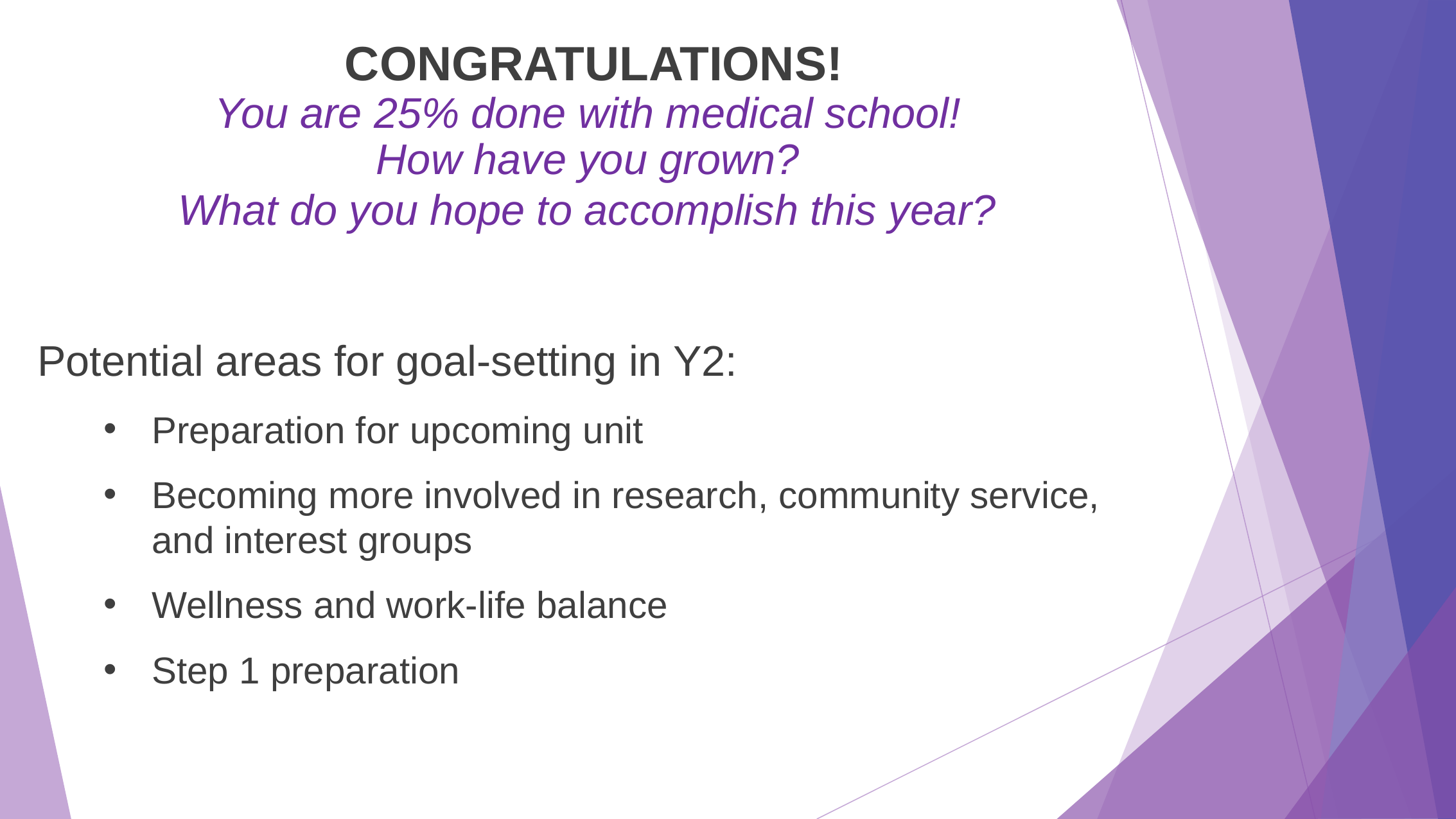

# GOAL SETTING IN Y2
CONGRATULATIONS!
You are 25% done with medical school!
How have you grown?
What do you hope to accomplish this year?
Potential areas for goal-setting in Y2:
Preparation for upcoming unit
Becoming more involved in research, community service, and interest groups
Wellness and work-life balance
Step 1 preparation

## Slide 5
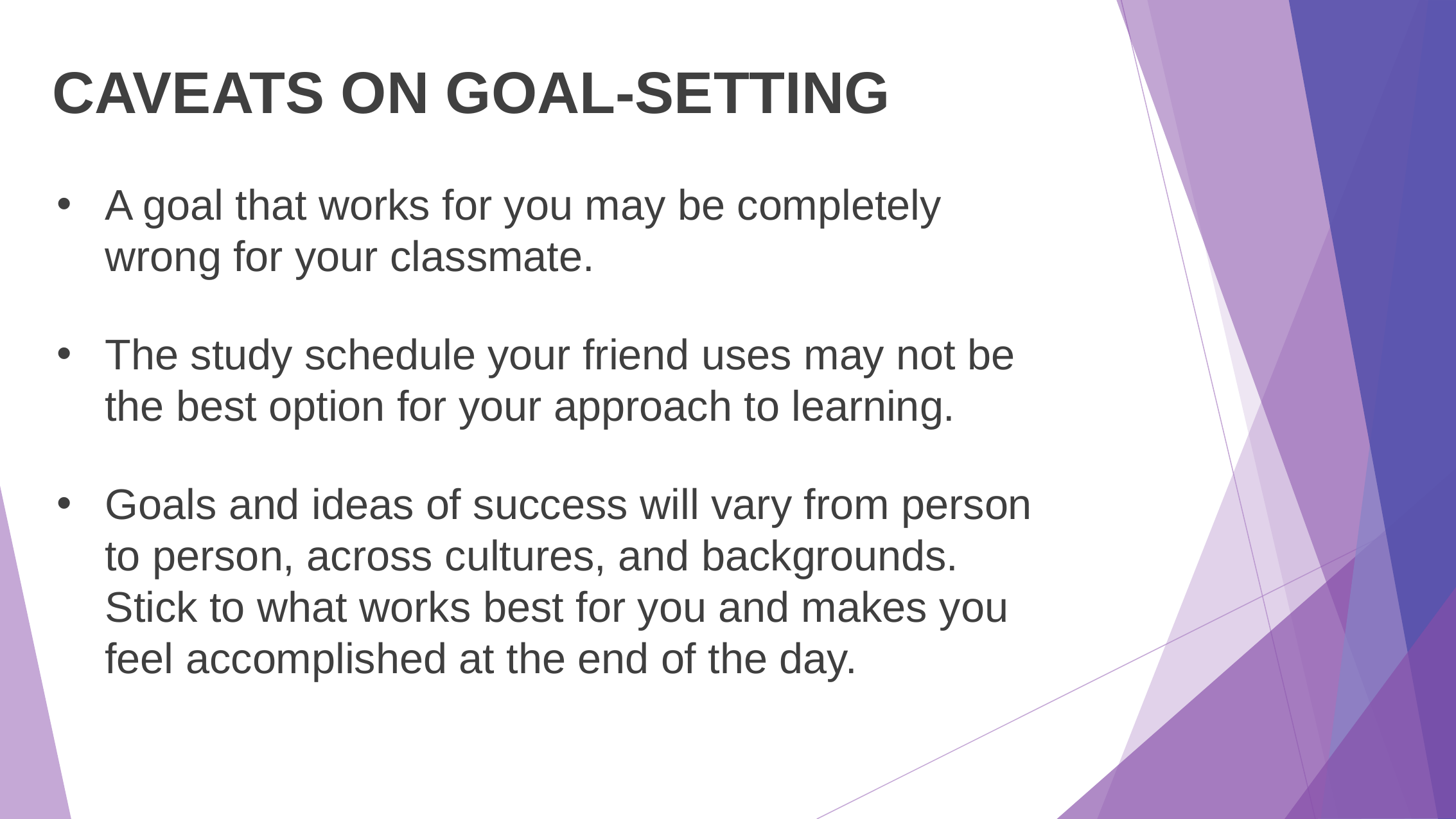

# CAVEATS ON GOAL-SETTING
A goal that works for you may be completely wrong for your classmate.
The study schedule your friend uses may not be the best option for your approach to learning.
Goals and ideas of success will vary from person to person, across cultures, and backgrounds. Stick to what works best for you and makes you feel accomplished at the end of the day.

## Slide 6
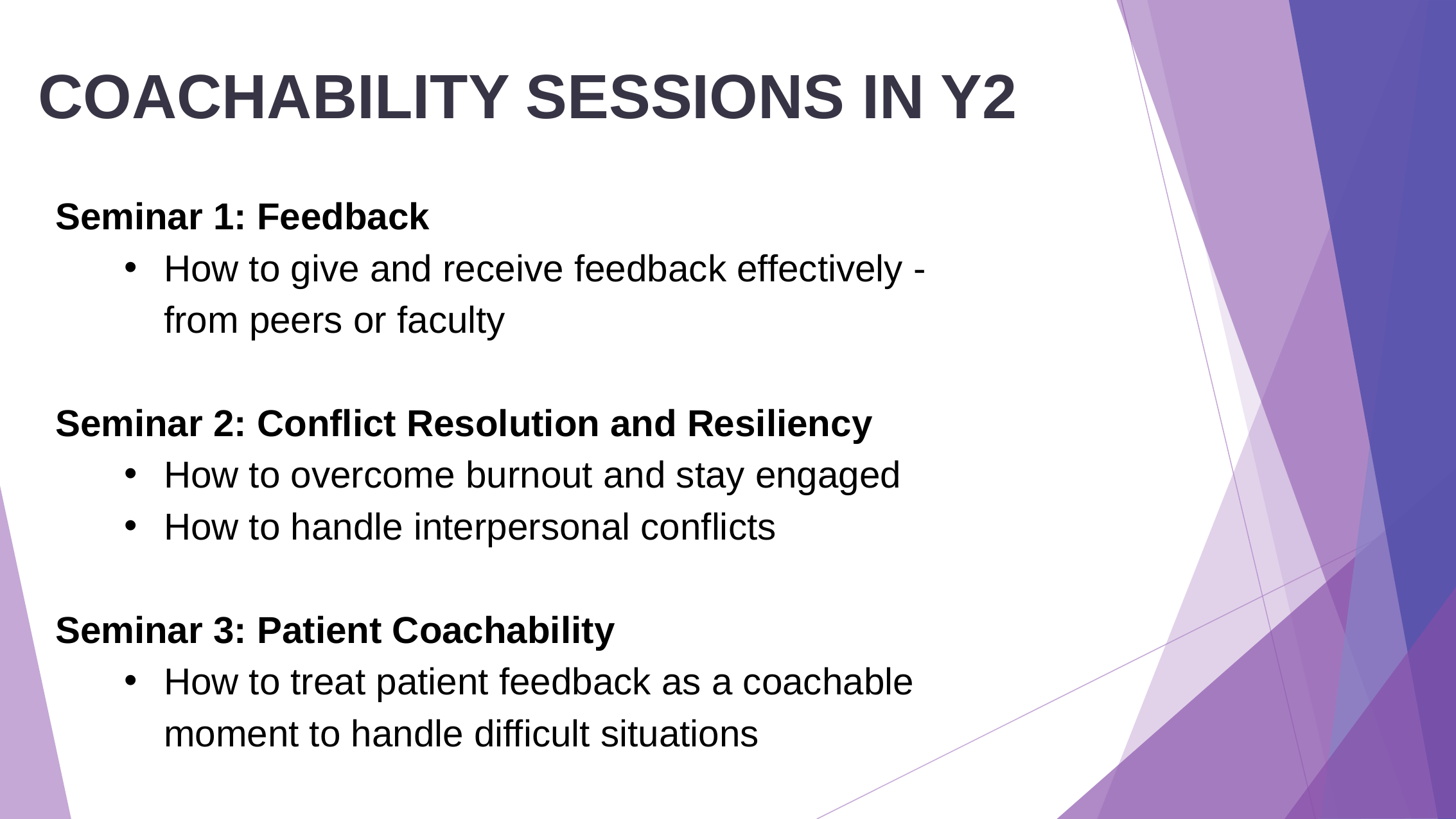

# COACHABILITY SESSIONS IN Y2
Seminar 1: Feedback
How to give and receive feedback effectively - from peers or faculty
Seminar 2: Conflict Resolution and Resiliency
How to overcome burnout and stay engaged
How to handle interpersonal conflicts
Seminar 3: Patient Coachability
How to treat patient feedback as a coachable moment to handle difficult situations

## Slide 7
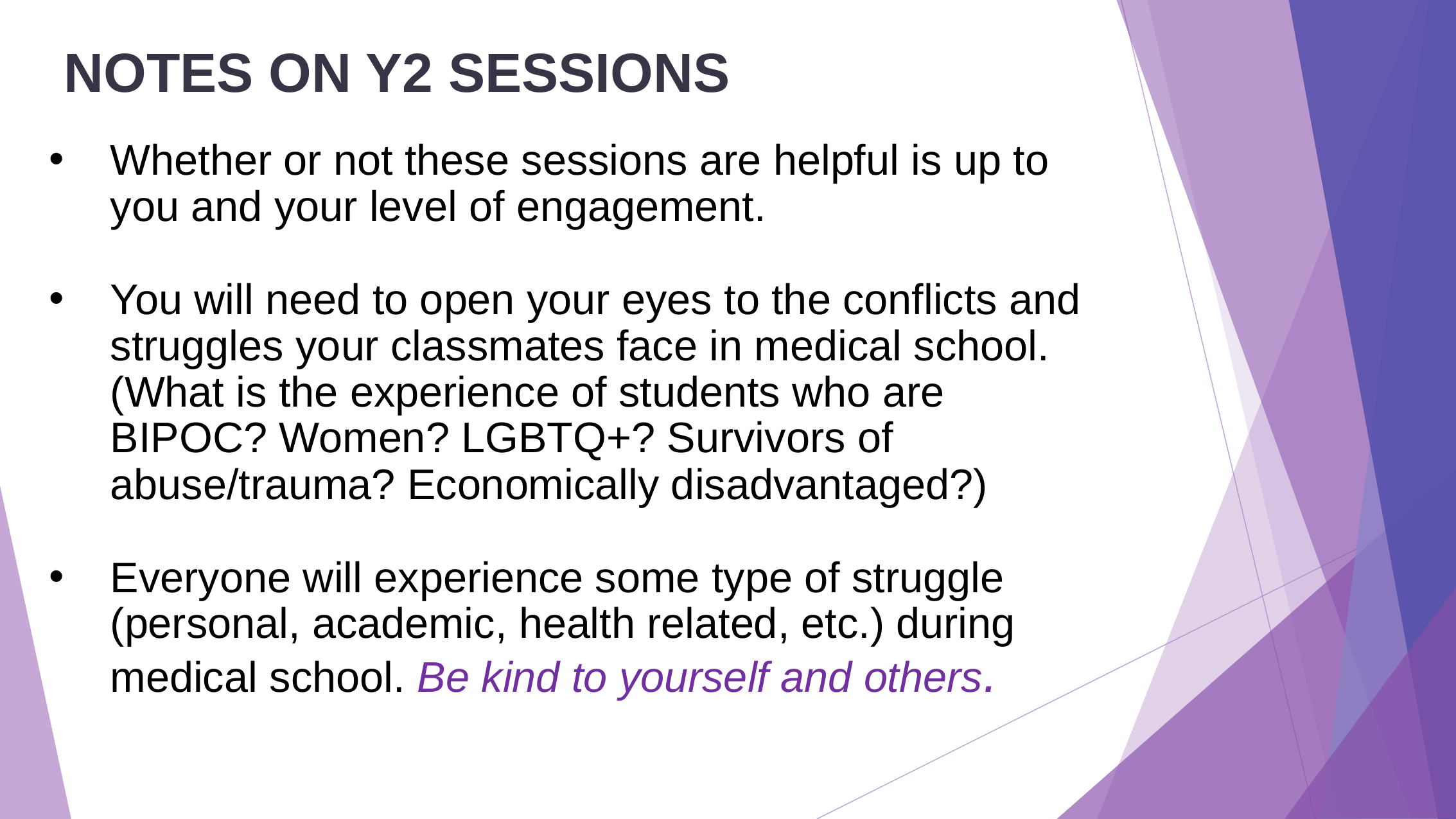

# NOTES ON Y2 SESSIONS
Whether or not these sessions are helpful is up to you and your level of engagement.
You will need to open your eyes to the conflicts and struggles your classmates face in medical school. (What is the experience of students who are BIPOC? Women? LGBTQ+? Survivors of abuse/trauma? Economically disadvantaged?)
Everyone will experience some type of struggle (personal, academic, health related, etc.) during medical school. Be kind to yourself and others.

## Slide 8
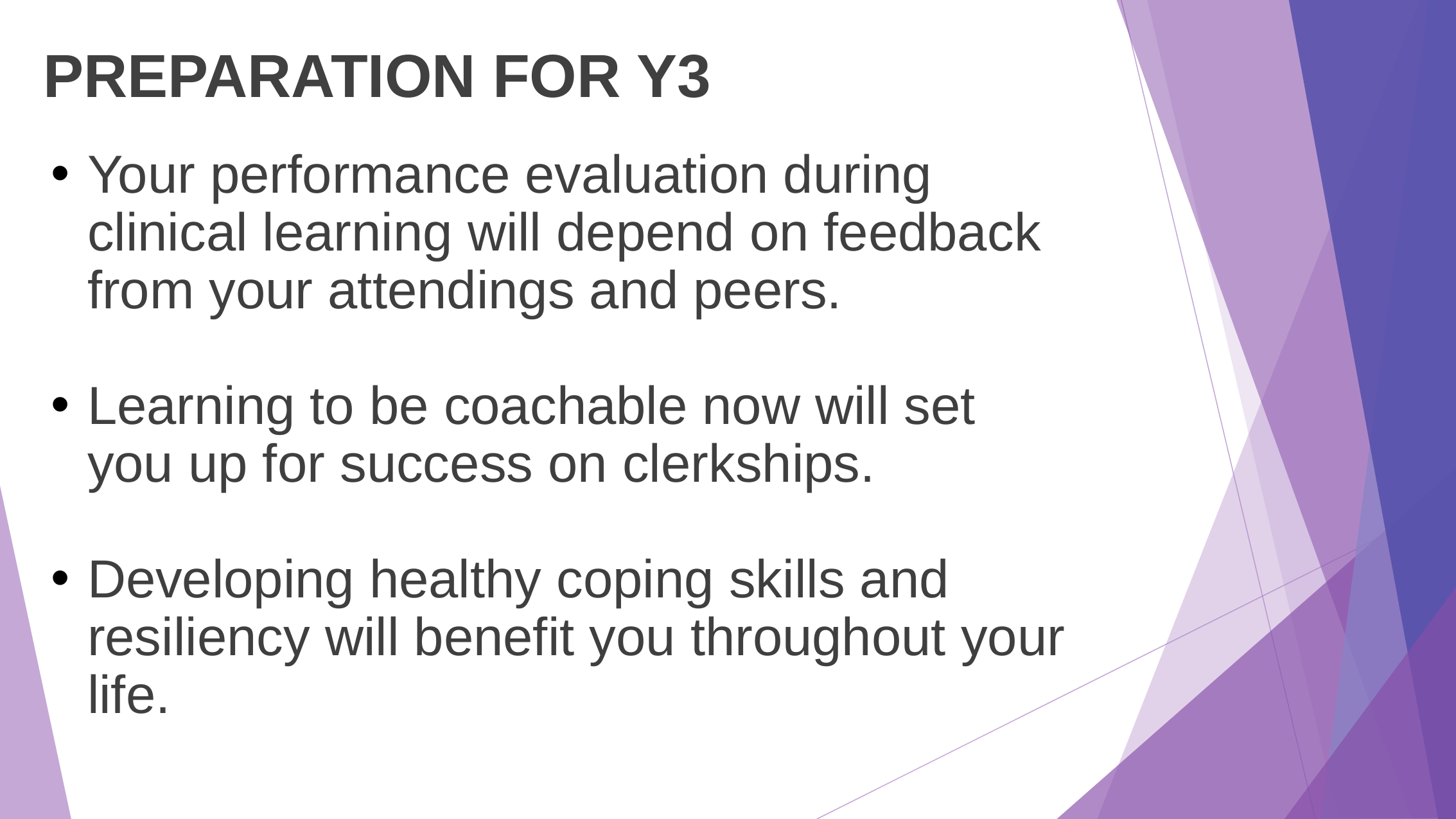

# PREPARATION FOR Y3
Your performance evaluation during clinical learning will depend on feedback from your attendings and peers.
Learning to be coachable now will set you up for success on clerkships.
Developing healthy coping skills and resiliency will benefit you throughout your life.

## Slide 9
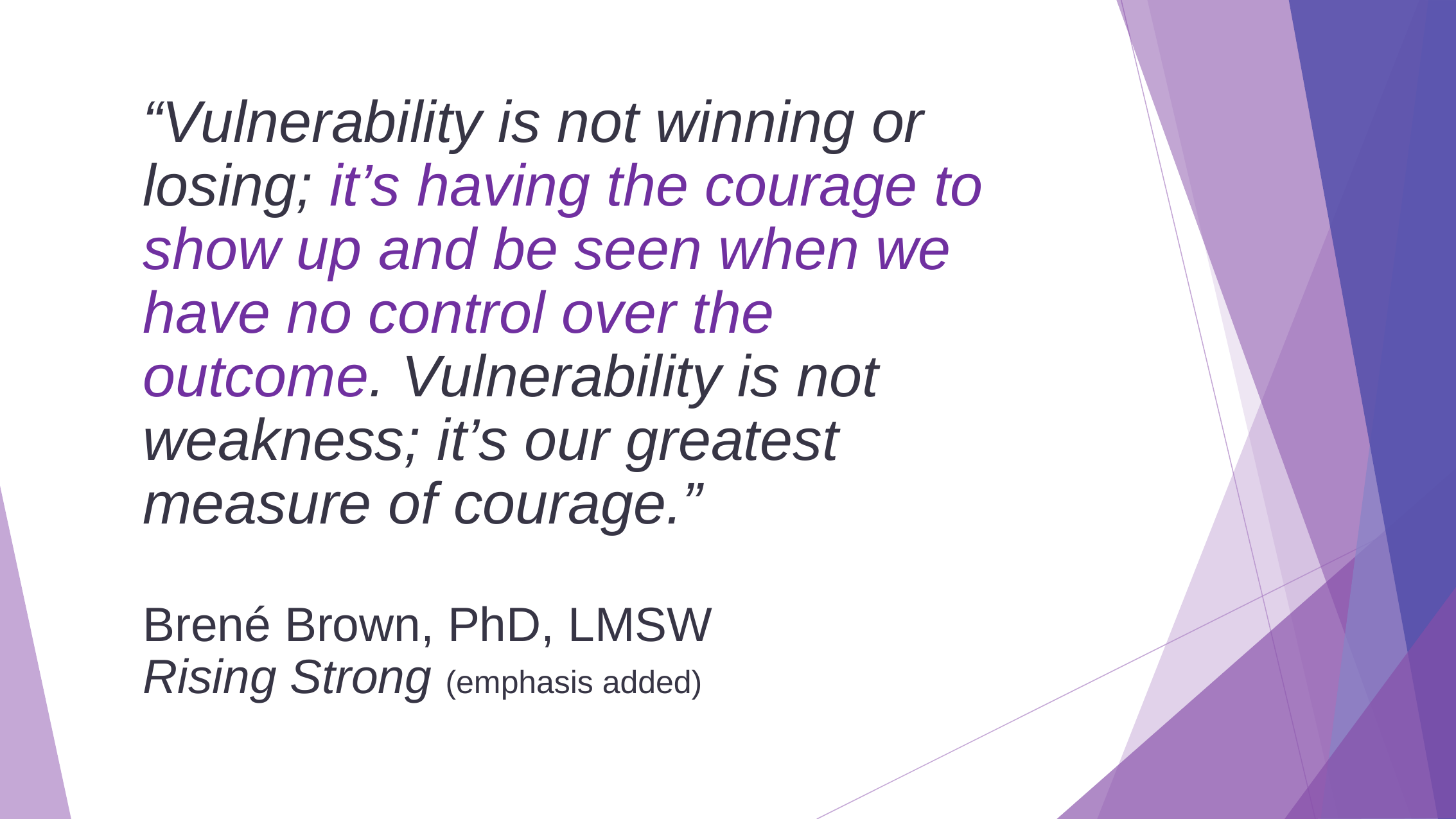

“Vulnerability is not winning or losing; it’s having the courage to show up and be seen when we have no control over the outcome. Vulnerability is not weakness; it’s our greatest measure of courage.”
Brené Brown, PhD, LMSW
Rising Strong (emphasis added)

## Slide 10
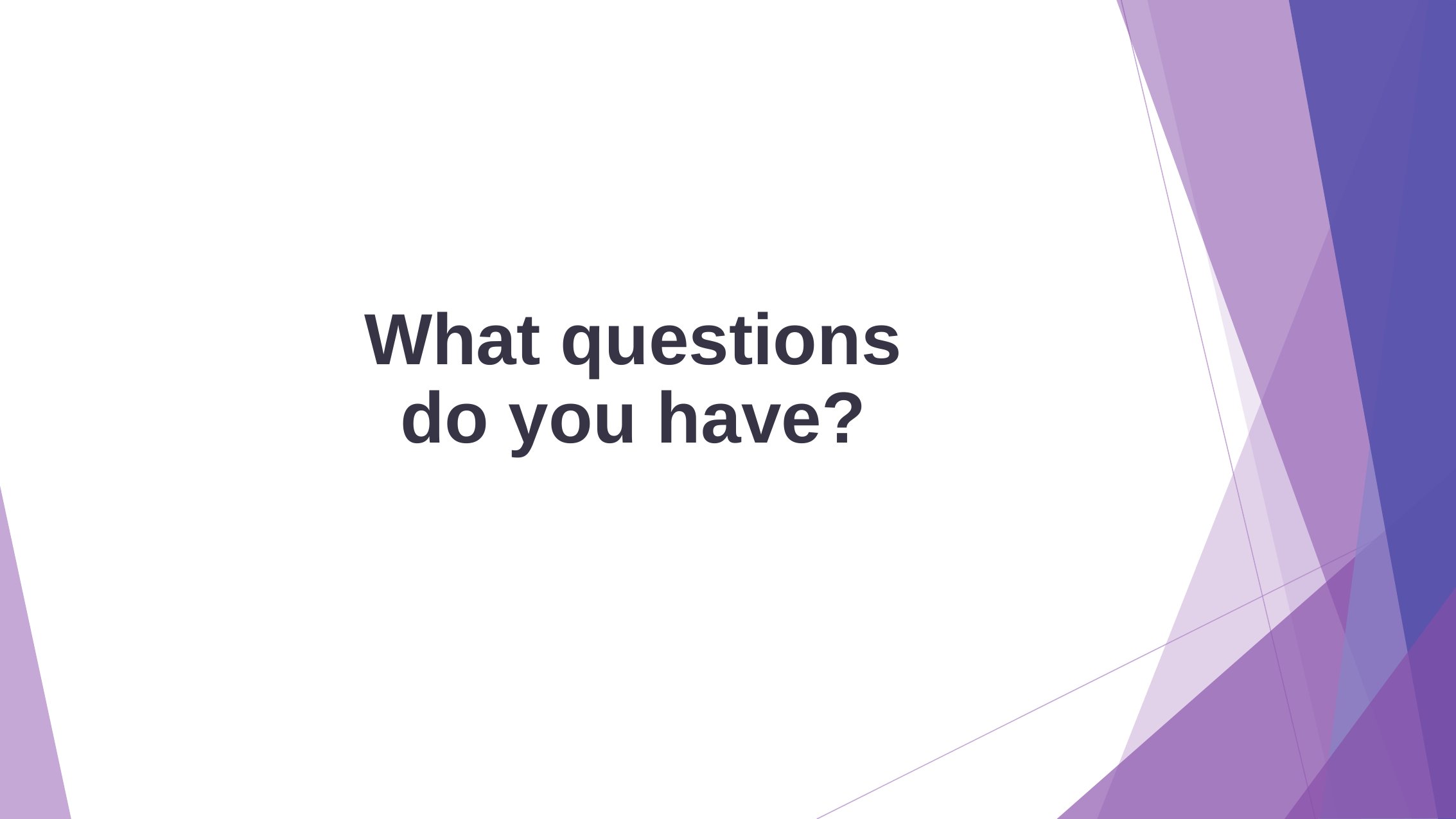

# What questions do you have?
