# Supplementary material for: Coachability: A Longitudinal Curriculum to Promote Medical Students’ Growth Mindset, Feedback Utilization, and Resilience
Source: MedEdPORTAL. 2024 Oct 11;20:11450. doi: 10.15766/mep_2374-8265.11450 (PMC11467082; doi:10.15766/mep_2374-8265.11450)
Supplement: Supplementary file 1 — Year 1 - Coachability.pptxYear 1 - Self-Assessment.docxYear 2 - Coachability.pptxSeminar 1 - Facilitator Guide.docxSeminar 2 - Facilitator Guide.docxSeminar 3 - Facilitator Guide.docxPostseminar Survey.docxFocus Group Protocol.docx [file mep_2374-8265.11450-s001.zip › D. Seminar 1 - Facilitator Guide.docx]

**Seminar 1 - Feedback - Facilitator Guide**

Seminar Objectives:

1. Conduct a reciprocal goal-setting discussion that facilitates the feedback process
2. Explain the value of specific feedback and the appropriate times to seek it
3. Demonstrate the ability to navigate unproductive and potentially threatening feedback

Seminar Preparation:

This seminar comprises three hands-on role-play drawing activities to help pre-clinical students navigate feedback experiences of varying quality. The seminar is ideally conducted in small groups of six students, and is amenable to either in-person format (preferred) or virtual format. Before an in-person seminar, print the role instructions for each activity (located at the end of this document) and cut them along the dotted lines for distribution to students. Before a virtual seminar, create separate role instructions files using the dotted lines as reference points. For each activity, two students will each receive one role—Student or Coach—while the other students observe.

Seminar Conduct:

Allow 15 minutes per activity (45 minutes total), splitting discussion time and drawing time evenly at first, and then adjusting to your group dynamic from there. If the seminar is conducted in-person, use whiteboards and markers or other shared visualization tools (e.g., computer and projector) to conduct the activities. If the seminar is conducted virtually, have someone in your small group create a Google Jamboard (or other online document-sharing app) and share it with everyone. At least some students in the small group will need to be comfortable using the drawing tools for the selected app. Regardless of format, we recommend assigning different students for each role-play activity so that everyone has a chance to participate actively.

**___________________________________________________________________________________**

**Activity 1 - Sharing Performance Goals and Expectations:**

Begin this activity by explaining to your small group that this role-play drawing activity is meant to simulate an environment where you are assigned a task and will be receiving feedback on your task performance. The two roles are Student and Coach.

**Activity 1 role instructions:**

Each role has different, hidden objectives for the simulation, as follows:

- Student: Your task is to draw a puppy. Please make your drawing as **DETAILED** and **REALISTIC** as possible. Your Coach will observe you and will give you feedback on your performance when your drawing is complete.
- Coach: Your task is to evaluate your Student drawing a puppy. Once they are finished drawing, please provide feedback to them on the **FOLLOWING CRITERIA ONLY**: # of times the eraser used/corrections are made, how many colors the Student used to make the drawing, how long the Student took to complete the drawing.

**Activity 1 discussion (for AFTER activity is complete):**

Ask the following questions of the Student, Coach, or Anyone in the small group:

- (Student) Please share with the group what your objective was.
- (Coach) Please share with the group what your objective was.
- (Student) Were you aware of how you were being evaluated?
  - How helpful was the Coach’s feedback in evaluating your goal of drawing a detailed and realistic puppy?
  - Would knowing how you were being evaluated changed how you achieved your objectives?
- (Coach) If the Student had told you up front that their goal was to draw a detailed and realistic puppy, would that have changed your feedback criteria?
- (Anyone in the small group) How would sharing the feedback criteria **beforehand** change this process? How would sharing your goals with your Coach change the outcome?
  - Who should share goal and feedback criteria information?
    - It is really a shared task - you have to inform your Coach what your goals are, and you should clarify with your Coach what their expectations are.
  - How and when do you have these goal-setting/expectations conversations?
    - Ideally you should clarify your goals and expectations any time you are working with someone new. After you have worked with the same person for a while, it is always beneficial to reevaluate goals and expectations as well.
  - How would you go about understanding expectations proactively?

*Make sure your small group understands the importance of sharing their goals (ideally balanced growth and performance-oriented goals) with their clinical preceptors ahead of time so that the preceptor has a framework to evaluate the student. The student should also clarify what the preceptor’s expectations are so that they can tailor their behaviors appropriately. Hopefully they can see from this activity that poor communication can lead to frustration and misunderstandings for students and preceptors. Strong, proactive communication can facilitate better learning opportunities.*

____________________________________________________________________________________

**Activity 2 – Seeking and Incorporating SPECIFIC Feedback:**

Begin by explaining that this activity is similar to the previous one in that there is again a Student and Coach combination, but this time the pair will be allowed to communicate. It is recommended to assign the Student and Coach roles to a different pair of students from Activity 1 to maximize the number of students in the group who get to play an active role in this seminar.

**Activity 2 role instructions:**

- Student: Your task is to **draw a tree.** You may ask for feedback from your Coach during the drawing process.
- Coach: Your task is to provide feedback to your Student, who is drawing a **palm tree.**
  - However, you may only provide **specific** information if your Student poses a **specific** question, such as: “Is this tree big enough?” “Am I using the correct colors?” “Are these leaves the right shape?”
  - If your Student asks you a **general** question, you must respond **generally**, such as: “Not bad for your first time.” “You’ll draw it better with more knowledge of trees (or more practice drawing).” “Well, you’re not supposed to be perfect at it as a 2nd year student.”

**Activity 2 discussion (for AFTER activity is complete):**

Ask the following questions of the Student, Coach, or Anyone in the small group:

- (Student) Please share with the group what your objective was.
- (Coach) Please share with the group what your objective was.
- (Student) How valuable is feedback like “Good job,” “You’ll get better,” “That was fine?”
- (Anyone in the small group) What can we do to receive more specific and valuable feedback from our Coaches?
  - If you ask “How did I do today?” which is a very general question, you will most likely get a general answer in return.
  - If you instead ask something specific like “What can I do to improve my case presentations?” you will likely get a more specific answer.
- (Anyone in the small group) When is an appropriate time to ask for feedback? The aim is to think of feedback as more of a check-in than a formal evaluation, and to seek feedback at regular, shorter intervals.
  - Any time your Coach asks “Do you have any questions?” it is probably a great time to ask for feedback.
  - Ask for specific feedback while performing a task. If you do this, you can incorporate the feedback as you go and improve your outcome. For example, if you are doing something in the OR, don’t just ask “Is this okay?” Be more specific. Instead you might ask “Am I holding this tool correctly? Is the stitch I just made too deep/not deep enough? I am having a hard time performing (insert task), can you make any recommendations about my technique?”

____________________________________________________________

**Activity 3 – Navigating Unproductive (Potentially Threatening) Feedback:**

Explain to your small group that this activity is similar in that feedback will be shared again, but this time at the discretion of the Coach. It is recommended to assign the Student and Coach roles to a different pair of students from Activities 1 and 2 to maximize the number of students in the group who get to play an active role in this seminar.

**Activity 3 role instructions:**

- Student: Your task is to **draw a house.** Your Coach will determine when to provide you with feedback.
- Coach: *<The Coach receives a reference house drawing.>* Please provide feedback to your Student on drawing a house that looks like this [the reference house]:
- When the Student has drawn something incorrectly, the **only** feedback you may give is “That is wrong.” or “No, not good.” Please announce this statement each time your Student draws something that does not align with the reference picture. Although your Student may ask for more detailed feedback, all you can answer is some version of “That is wrong.” (e.g., “That’s just not right.” “It’s incorrect.”). If nothing is wrong with your Student’s picture, you can say something like “I don’t know what else to tell you.” “Hmmm..” or shrug your shoulders.

NOTE - This activity should get aggravating—either the student will respond in a positive way, “I understand this is wrong, but what can I be doing better?” or they may respond negatively, such as by shutting down, rolling eyes, sighing, lashing out, etc. Either way of responding will generate the desired discussion.

**Activity 3 discussion (for AFTER activity is complete):**

- (Student) Please share with the group what your objective was.
- (Coach) Please share with the group what your objective was.
- (Student) How could you have received that feedback better?
- (Anyone in the small group) What are some reasons you might resist feedback you’ve gotten?
- (Anyone in the small group) How does it feel to only receive negative feedback?
- (Anyone in the small group) What should you do if you receive feedback you don’t agree with?
  - Most of the time-- brush it off, learn from it, and try to move on.

*Note: Attitude can be a barrier to feedback (this includes attitude of the student and attitude of the coach). If you have a poor attitude about the feedback you are getting, do not expect high quality, constructive feedback. If you realize your attending has a bad attitude about feedback, then you might need to use other skills to try and elicit quality, constructive feedback.*

- (Anyone in the small group) What other barriers to feedback can you think of?
- Miscommunication: Think back to the first activity about communicating goals and clarifying expectations.
- Fear of hurting someone’s feelings.
- Have you ever given dishonest feedback because you did not want to hurt a friend/family member’s feelings?
- Do you think our preceptors might do the same?

*Remind your small group that clerkship students will also be asked to give feedback (on attendings, residents, peers). In so doing, they should remember how it feels to get only vague feedback and/or negative feedback and try to give honest feedback that will lead to improvements! Constructive feedback is important for everyone.*

**Seminar 1 - Feedback - Learner Instructions**

*Cut along the dotted lines and distribute EACH set of role instructions to ONE learner.*

*Instruct the role-players not to share their instructions with each other or the other learners in the small group.*

**----------------------------------------------------------------------------------------------------------------------------**

**Activity 1 Student Instructions**

Your task is to draw a puppy. Please make your drawing as **DETAILED** and **REALISTIC** as possible. Your Coach will observe you and will give you feedback on your performance when your drawing is complete.

**----------------------------------------------------------------------------------------------------------------------------**

**Activity 1 Coach Instructions**

Your task is to evaluate your Student drawing a puppy. Once they are finished drawing, please provide feedback to them on the **FOLLOWING CRITERIA ONLY**:

# of times the eraser used/corrections are made

How many colors the Student used to make the drawing

How long the Student took to complete the drawing.

**Seminar 1 - Feedback - Learner Instructions, Continued**

*Cut along the dotted lines and distribute EACH set of role instructions to ONE learner.*

*Instruct the role-players not to share their instructions with each other or the other learners in the small group.*

**----------------------------------------------------------------------------------------------------------------------------**

**Activity 2 Student Instructions**

Your task is to **draw a tree.** You may ask for feedback from your Coach during the drawing process.

**----------------------------------------------------------------------------------------------------------------------------**

**Activity 2 Coach Instructions**

Your task is to provide feedback to your Student, who is drawing a **palm tree.**

However, you may only provide **specific** information if your Student poses a **specific** question, such as: “Is this tree big enough?” “Am I using the correct colors?” “Are these leaves the right shape?”

If your Student asks you a **general** question, you must respond **generally**, such as: “Not bad for your first time.” “You’ll draw it better with more knowledge of trees (or more practice drawing).” “Well, you’re not supposed to be perfect at it as a 2nd year student.”

**Seminar 1 - Feedback - Learner Instructions, Continued**

*Cut along the dotted lines and distribute EACH set of role instructions to ONE learner.*

*Instruct the role-players not to share their instructions with each other or the other learners in the small group.*

**----------------------------------------------------------------------------------------------------------------------------**

**Activity 3 Artist Instructions**

Your task is to **draw a house.** Your Coach will determine when to provide you with feedback.

**----------------------------------------------------------------------------------------------------------------------------**

**Activity 3 Coach Instructions**

Please provide feedback to your Student, how is attempting to draw a house that looks like this:


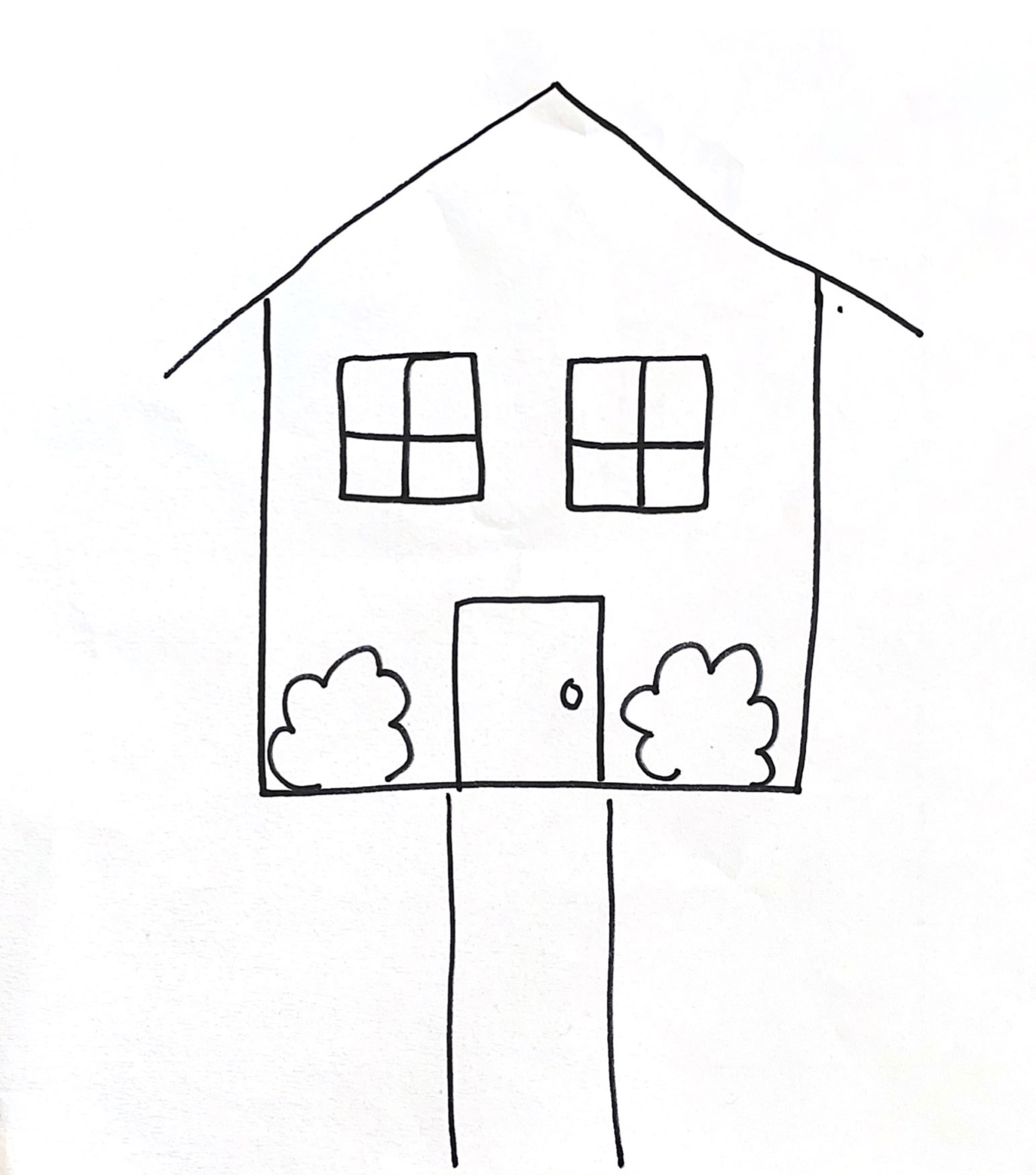
 *Author owned image.*

When the Student has drawn something incorrectly, the **only** feedback you may give is “That is wrong.” or “No, not good.” Please announce this statement each time your Student draws something that does not align with the reference picture. Although your Student may ask for more detailed feedback, all you can answer is some version of “That is wrong.” (e.g., “That’s just not right.” “It’s incorrect.”). If nothing is wrong with your Student’s picture, you can say something like “I don’t know what else to tell you.” “Hmmm..” or shrug your shoulders.
