# Supplementary material for: Coachability: A Longitudinal Curriculum to Promote Medical Students’ Growth Mindset, Feedback Utilization, and Resilience
Source: MedEdPORTAL. 2024 Oct 11;20:11450. doi: 10.15766/mep_2374-8265.11450 (PMC11467082; doi:10.15766/mep_2374-8265.11450)
Supplement: Supplementary file 1 — Year 1 - Coachability.pptxYear 1 - Self-Assessment.docxYear 2 - Coachability.pptxSeminar 1 - Facilitator Guide.docxSeminar 2 - Facilitator Guide.docxSeminar 3 - Facilitator Guide.docxPostseminar Survey.docxFocus Group Protocol.docx [file mep_2374-8265.11450-s001.zip › E. Seminar 2 - Facilitator Guide.docx]

**Seminar 2 - Conflict Resolution and Resiliency – Facilitator Guide**

Seminar Objectives:

1. Self-assess your level of burnout
2. Identify common factors that cause burnout
3. Describe the role of interpersonal conflict in causing burnout
4. Apply strategies for resolving or overcoming interpersonal conflicts
5. Describe sources of your own burnout and how burnout may impact your work
6. Identify strategies for preventing burnout
7. Articulate ways to overcome burnout and build resiliency

Seminar Preparation:

1. Print a copy of the *burnout survey* (adapted from the Maslach Burnout Inventory-Student Survey, appended below) for each student participating in the seminar. Alternatively, the official Maslach survey is commercially available for purchase at <https://www.mindgarden.com/313-mbi-general-survey-for-students>.
2. Print one set of the *common factors causing burnout* (appended below). Cut along the dotted lines to form cards.
3. Print a copy of this *facilitator guide*.
4. Create a list of contact information for individuals that may be able to provide support for students at your institution (examples may include Dean or other administration, mental health counselors, etc.)

Seminar Conduct:

This discussion-based seminar is conducted in three parts that collectively take about 60 minutes: *Understanding Your Burnout*, *Interpersonal Conflict as a Source of Burnout*, and *Building Resiliency*. The seminar is ideally conducted in small groups of six students, and is amenable to either in-person format (preferred) or virtual format.

Facilitator Instructions and Guidance:

**Seminar Part 1: Understanding Your Burnout**

1. Before diving into a discussion of burnout, ask the students: “What is causing you the most stress right now? How are you currently, if at all, coping with that stress?” (3 min)
2. Distribute a copy of the *burnout survey* to each student and allow them to fill it out on their own. (5-6 minutes).
3. Reflect collaboratively with the students on their burnout survey results. (7-8 min)
   1. Ask the students:
      1. What results did you find surprising?
      2. Before taking this, did you consider yourself to be experiencing burnout? Do your results match what you thought?
      3. High point stressors on your list?
      4. Things that aren’t as challenging for you? Why do you think that is?
4. Facilitate discussion on the *common factors causing burnout* (5-6 min)
   1. There are many factors that contribute to burnout and it would take a long time to create an exhaustive list. The coachability team retrospectively created a list of what we saw as common factors that contribute to burnout over the continuum of medical school. With the help of an article by Mayo Clinic about workplace burnout we have identified 7 main areas of interest:
      1. Lack of control
      2. Unclear expectations
      3. Dysfunctional study space/workplace environment
      4. Low job satisfaction
      5. Extremes of activity
      6. Lack of social support
      7. Work-life imbalance
   2. At this time, read off the *common factors causing burnout* cards we have created and lay them on the table for the students to see. Ask the students:
      1. Does this list resonate with you?
      2. Is there anything major that you feel is missing and would like to add?
5. Facilitate a discussion of burnout and its influence on learning/being coachable (2-3 min)
   1. Ask: So why do we care? What does this even have to do with being coachable? Well think back to the list we made on the board about what makes an effective learner.
      1. Energetic, eager, able to process new information, able to focus on task at hand, be engaged in clinical environment, etc.
   2. Ask:
      1. If you are feeling burned out what are the chances you will fit the characteristics of an effective learner?
      2. If someone gives you feedback, how open would you be to accepting and using it?
      3. If someone is trying to coach you through something new, how engaged would you be able to be in the activity/information provided?
   3. Ask: Does it make sense how burnout can affect your ability to learn and be coachable?

____________________________________________________________________________________

**Seminar Part 2: Interpersonal Conflict as a Source of Burnout**

1. Activity: Reflect and respond to medical student experience (5 min)
   1. Read the following scenario to the small-group: *You have started on your first week of internal medicine inpatient wards as a clerkship student. You have several other clerkships under your belt, and you are still getting a feel for the flow of rounds, note writing, and other responsibilities. After morning rounds, the senior resident on your team assigns you a topic to present to the group after lunch. You aren’t very familiar with the topic and need some time to read more about it. During your lunch hour there is a mandatory lecture for medical students, leaving you with little time to prepare. You mention this to your senior, but you notice that they seem overwhelmed. After approaching them to talk about it, they tell you that they “don’t have time for this” and leave the room.*
   2. Facilitate reflective discussion on the scenario, using these open-ended questions:
      1. Have you ever been in a situation like this before?
      2. How would you approach this situation?
      3. What should you do if problems continue?
2. Facilitate a group discussion on how to work through interpersonal conflict (5-6 min)
   1. Ask: What is the goal of conflict resolution?
      1. To have conversations of understanding
   2. Ask: Why is this important?
      1. Reduces tension in relationship
      2. Creates a collaborative and safe environment
      3. Unresolved conflict affects future interactions and increases the likelihood that other conflicts will arise
      4. Prevents burnout long-term (*highlight that this is why we are having discussions on these topics hand-in-hand in this seminar)*
3. Ask: If you had to explain how to work through conflict how would you break it down? (5-6 min)
   1. Steps to successfully negotiating resolution of conflict:
      1. Listen to understand the conflict and start w/ curiosity
         1. Be sure to ask clarifying questions and aim to foster mutual sense of agency
         2. As a student, you are allowed to be curious because you are the learner. Use that to your advantage when trying to understand the conflict.
         3. Be sure to be self-reflective about how you could have contributed to the situation as well.
      2. Communicate
         1. It is important to understand that people are hardwired to process physical and social threats the same way. Some may deal with these situations through anger, detachment, avoidance, etc. Therefore, be aware of your tone and choice of words.
      3. Brainstorm possible resolutions
      4. Choose the best resolution or compromise
      5. Use a third-party mediator if necessary and explore alternatives
4. Revisit the original scenario with these strategies: *Go around the group and ask each person to name one strategy that could be applied to help resolve the conflict.* (5 min)

____________________________________________________________________________________

**Part 3: Building Resiliency**

1. Ask: Can you think of an example in your past when you have successfully coped with stress? What did you do at that time? (3 min)
2. Ask: I know we already asked this question, but this time I really want you to self-reflect. What is causing you the most stress right now? (3 min) (If it’s Step 1, be more specific. What is it about Step 1 that is getting to you?)
   1. Try to encourage self-reflection and identifying underlying causes of stress (i.e. malignant study environment, getting behind schedule/procrastination, inability to balance Step 1 with unit, overcommitted to extracurricular/outside activity, etc.)
3. Facilitate collaborative reflection: Based on the common factors that contribute to burnout, or what you have identified as a source of your own stress, how could we modify these factors to reduce our risk of burnout in the medical school setting? -- *Encourage students to come up with their own ideas first, offer suggestions from this list only when their ideas are exhausted*. (5-10 min)
   1. What kinds of things can you do to address this feeling of “lack of control?”
      1. Create and stick to a schedule. Include time off into your schedule.
         1. Gives you the chance to monitor and see progress.
         2. Should help manage the urge to procrastinate.
      2. Align your goals with what you need to accomplish
      3. Acceptance: you will often not be able to control your schedule and things will unexpectedly come up. Try to be as flexible as possible.
         1. Include time in your schedule to “catch up” on things you didn’t get to for whatever reason
   2. Unclear job expectations
      1. Discuss expectations and goals at the beginning of a mentor session/tutor group
      2. Reach out to students who have done the job/task before for guidance.
   3. Dysfunctional workplace dynamic
      1. Know who to turn to if you are put in very uncomfortable situations- i.e. nurse educator in charge of clerkship, tutor group leader, 4th year mentors.
      2. Try to not contribute to the negative environment.
      3. Speak up if you feel things are not working well
   4. Lack of job satisfaction
      1. Revisit what you really enjoy about the field of medicine, remind yourself why you wanted to go to medical school in the first place
      2. Dedicate time to connecting with/or refocusing your time/energy on these things
   5. Extremes of activity
      1. Create a realistic schedule and stick to it. If you’re feeling unfocused, don’t waste your time -- take a break and come back to it. Work on procrastination tendencies
      2. If chaotic environment/overwhelmed with schedule, prioritize and focus on one task at a time.
   6. Lack of social support
      1. Reach out to friends in class, on rotation with you, residents, even attendings. Chances are if you are stressed, they are too.
      2. Open up to someone outside of medicine- family members, other friends because even if they do not understand exactly what you are going through they can often offer fresh perspectives (plus they offer an easier opportunity to get away from and turn of your medical brain for a bit)
      3. Do your best to focus on the opportunities medicine has opened from you and focus less on the things you feel you have had to sacrifice to be here
   7. Work-life imbalance
      1. Pick one or 2 things important to you outside of medicine and commit to doing them regularly, GUILT FREE
      2. Set realistic goals to accomplish for the day
      3. Make time for the people who are important to you
4. Debrief the students: *The Coachability team wants you to be aware that we don’t expect that you will leave today with all of your burnout problems solved as the most resilient medical students ever to walk these halls, but we do hope that today helped you realistically identify what is contributing the greatest to your burnout and how you might start managing it better. We also hope you will be able to better identify burnout when it starts to creep into your day to day life.*
5. Provide *list of contact information* to students of resources at your institution when they are feeling overwhelmed.

**Burnout Survey**

*(adapted from the Maslach Burnout Inventory-Student Survey cited in the appendix of Shi et al.*)*

Rank each statement using the scale below. Higher scores indicate more burnout.

**Less Burnout**

1 = Never

2 = A few times a year or less

3 = Once a month or less

4 = A few times a month

5 = Once a week

6 = A few times a week

7 = Every day

**More Burnout**

**Exhaustion**

1. I feel emotionally drained by my studies. _____
2. I feel used up at the end of a day at medical school. _____
3. I feel tired when I get up in the morning and I have to face another day at medical school _____
4. Studying or attending a class is really a strain for me. _____
5. I feel burned out from my studies. _____

**Cynicism**

1. I have become less interested in my studies since my enrollment in medical school. _____
2. I have become less enthusiastic about my studies. _____
3. I have become more cynical about the potential usefulness of my studies. _____
4. I doubt the significance of my studies. _____

**Professional Efficacy**

1. I can effectively solve the problems that arise in my studies. _____
2. I believe that I make an effective contribution to the classes/tutor group I attend. _____
3. In my opinion, I am a good student. _____
4. I feel stimulated when I achieve my study goals. _____
5. I have learned many interesting things during the course of my studies. _____
6. During class, I feel confident that I am effective and getting things done. _____

*Shi Y, Gugiu PC, Crowe RP, Way DP. A Rasch Analysis Validation of the Maslach Burnout Inventory–Student Survey with Preclinical Medical Students. *Teaching and Learning in Medicine*. 2018;31(2):154-169.

**Common Factors Causing Burnout**

*Cut along the dotted lines to make cards, and distribute a set of cards to each student.*

**---------------------------------------------------------------------------------------------------**

**Lack of Control**

1. Lecture content that is disorganized or at an inappropriate level of depth
2. Lecture content that seems irrelevant to what you need or want to know
3. Scheduling inconveniences (personal meetings, special occasions, etc.)
4. Outsized importance of Step 1 exam performance

**---------------------------------------------------------------------------------------------------**

**Unclear Job Expectations**

1. Uncertainty regarding how to balance Step 1 studying with studying for curricular exams
2. Vague preceptor expectations in mentored clinical experiences
3. Uncertainty regarding what will be on unit tests/clinical exams, etc.

**---------------------------------------------------------------------------------------------------**

**Dysfunctional Workplace Dynamic**

1. Negative interpersonal context within learning groups
2. Pressure from classmates
3. Negative comparison of self to classmates
4. Preceptors, faculty, other students you do not see eye to eye with

**---------------------------------------------------------------------------------------------------**

**Poor Job Satisfaction**

1. Disconnect between what you perceived medical school would be like and what it has actually been like
2. Feeling like you don’t have access to or time to focus on the things that made you want to come to medical school in the first place (patient interactions, certain volunteer activities, etc.)

**---------------------------------------------------------------------------------------------------**

**Extremes of Activity**

1. Overwhelming study schedule
2. Lack of sleep
3. Uncontrollable swings in level of stimulation: finding yourself feeling overwhelmed with not enough time to complete your to-do-list, to situations where you feel under stimulated and bored (and possibly worrying about everything you should/want to be getting done instead)

**---------------------------------------------------------------------------------------------------**

**Lack of Social Support**

1. Feeling like you cannot relate to classmates or fearing inadequacy in the eyes of other classmates
2. Not feeling like you can open up to family or friends about stressors, or maybe you do but it feels like they don’t “get it”
3. Feeling “stuck” or left out when you see non-medical peers engaging in life experiences you have sacrificed to attend to your medical education

**----------------------------------------------------------------------------------------------------------------------------**

**----------------------------------------------------------------------------------------------------------------------------**

**Work-Life Imbalance**

1. Having to devote free time to studying or other school-related activities
2. Feeling guilt for not studying
3. Not attending to important relationships in your life (parents, close friends, partners, grandparents, etc.)
4. Rarely making the things you love to do a priority

**----------------------------------------------------------------------------------------------------------------------------**
